# Raptor mediates the selective inhibitory effect of cardamonin on RRAGC-mutant B cell lymphoma

**DOI:** 10.1186/s12906-023-04166-7

**Published:** 2023-09-26

**Authors:** Ying Liu, Yanting Zhu, Huajiao Chen, Jintuo Zhou, Peiguang Niu, Daohua Shi

**Affiliations:** Department of Pharmacy, College of Clinical Medicine for Obstetrics & Gynecology and Pediatrics, Fujian Medical University Fujian Maternity and Child Health Hospital, 18 Daoshan Road, Fuzhou, 350001 Fujian China

**Keywords:** Raptor, Cardamonin, RagC, mTORC1, Follicular lymphoma

## Abstract

**Background:**

mTORC1 (mechanistic target of rapamycin complex 1) is associated with lymphoma progression. Oncogenic RRAGC (Rag guanosine triphosphatase C) mutations identified in patients with follicular lymphoma facilitate the interaction between Raptor (regulatory protein associated with mTOR) and Rag GTPase. It promotes the activation of mTORC1 and accelerates lymphomagenesis. Cardamonin inhibits mTORC1 by decreasing the protein level of Raptor. In the present study, we investigated the inhibitory effect and possible mechanism of action of cardamonin in RRAGC-mutant lymphoma. This could provide a precise targeted therapy for lymphoma with RRAGC mutations.

**Methods:**

Cell viability was measured using a cell counting kit-8 (CCK-8) assay. Protein expression and phosphorylation levels were determined using western blotting. The interactions of mTOR and Raptor with RagC were determined by co-immunoprecipitation. Cells overexpressing RagC wild-type (RagC^WT^) and RagC Thr90Asn (RagC^T90N^) were generated by lentiviral infection. Raptor knockdown was performed by lentivirus-mediated shRNA transduction. The in vivo anti-tumour effect of cardamonin was assessed in a xenograft model.

**Results:**

Cardamonin disrupted mTOR complex interactions by decreasing Raptor protein levels. RagC^T90N^ overexpression via lentiviral infection increased cell proliferation and mTORC1 activation. The viability and tumour growth rate of RagC^T90N^-mutant cells were more sensitive to cardamonin treatment than those of normal and RagC^WT^ cells. Cardamonin also exhibited a stronger inhibitory effect on the phosphorylation of mTOR and p70 S6 kinase 1 in RagC^T90N^-mutant cells. Raptor knockdown abolishes the inhibitory effects of cardamonin on mTOR. An in vivo xenograft model demonstrated that the RagC^T90N^-mutant showed significantly higher sensitivity to cardamonin treatment.

**Conclusions:**

Cardamonin exerts selective therapeutic effects on RagC^T90N^-mutant cells. Cardamonin can serve as a drug for individualised therapy for follicular lymphoma with RRAGC mutations.

**Supplementary Information:**

The online version contains supplementary material available at 10.1186/s12906-023-04166-7.

## Introduction

Follicular lymphoma (FL) is the second most common indolent non-Hodgkin lymphoma (NHL). It has a relapsing-remitting disease course with the risk of transformation into diffuse large B cell lymphoma (DLBCL) [1]. Chemoimmunotherapy is the main treatment for FL; however, the toxicity associated with therapeutic drugs negatively affects the quality of life of most patients [2]. With increasing research into the mechanisms underlying lymphomagenesis, targeted therapy has gradually attracted attention in recent years [3].

Mechanistic target of rapamycin complex 1 (mTORC1) regulates cellular homeostasis and metabolism by integrating environmental cues, including nutrient, oxygen, and growth factors. Dysregulation of mTORC1 is associated with the progression of several diseases, such as cancer, type 2 diabetes, and aging [4, 5]. mTORC1 is composed of mTOR, Raptor (regulatory protein associated with mTOR), mLST8 (mammalian lethal with Sect. 13 protein 8), proline-rich AKT substrate 40 kDa (PRAS40), and DEP-domain-containing mTOR-interacting protein (DEPTOR). Raptor is the core component of mTORC1 and is essential for the activation of mTORC1 [6]. Raptor recruits mTORC1 substrates, such as p70 S6 kinase 1 (S6K1) and induces their phosphorylation [7]. Raptor deletion decreases the activity of mTOR signalling and the proliferation and growth of cancer cells [8].

Upon stimulation with amino acids, mTORC1 translocates to the lysosomal surface, where it is activated. This translocation process requires the cooperation of Rag guanosine triphosphatase (Rag GTPase) heterodimers. There are four types of Rag GTPases in mammalian cells: RagA, RagB, RagC and RagD. Rag heterodimers consist of two functionally equivalent pairs: RagA·RagC, and RagB·RagD [9]. Depending on the nucleotide-binding states, Rag heterodimers are divided into “inactive” and “active” forms. Only when RagA or RagB binds to guanosine triphosphate (GTP) and RagC or RagD binds to guanosine diphosphate (GDP) do they interact with mTORC1 [9, 10]. The nucleotide-binding state of RagC (also known as RRAGC) is affected by mutations in its structural amino acids. RRAGC mutations cluster around nucleotide-binding sites, which results in an enhanced affinity of RagC for GDP [11, 12]. Subsequently, the interaction between the Rag GTPase heterodimer and mTOR increases, leading to mTORC1 activation [11].

Recent studies revealed that Rag GTPases recruit mTORC1 to lysosomes by directly binding to Raptor. The cryo-electron microscopy assay demonstrates that Raptor recognises the nucleotide states of Rag GTPases and inserts into the space between “active” Rag heterodimers to form a stable activating complex following mTOR activation [13, 14]. Zhang et al. identified recurrent oncogenic RRAGC mutations in approximately 10% of patients with FL [12]. In RRAGC-mutant DLBCL cells, the interaction between the Rag GTPase heterodimer and Raptor is enhanced, and the activation of mTORC1 is promoted even in the absence of amino acids [11]. RagC mutations accelerate lymphomagenesis and drive DLBCL sensitivity to pharmacological inhibition of mTOR [15].

Rapamycin and its derivatives (rapalogs) including everolimus and temsirolimus are classical mTOR inhibitors. Rapalogs inhibit mTOR activation by binding to mTOR with the assistance of FK506-binding protein 12 (FKBP12). Everolimus has therapeutic effects in certain types of NHL [16]. However, the clinical application of rapalogs is limited by adverse reactions and immunosuppressive effects [17]. Amino acid mutations in the mTOR protein have led to the emergence of mTOR inhibitor resistance [18]. This prompted the development of novel mTOR inhibitors.

Cardamonin is a natural chalcone derived from the seeds of *Amomum subulatum* [19]. Numerous studies have demonstrated the therapeutic effects of cardamonin in various cancers. It inhibits cell growth and proliferation, and induces apoptosis by modulating multiple molecular signalling pathways [20]. Our previous studies have revealed that the anti-tumour effect of cardamonin is associated with the mTOR signalling pathway [21–23]. Furthermore, unlike everolimus, cardamonin inhibited mTOR by decreasing the protein level of Raptor [24].

In this study, we evaluated the inhibitory effects of cardamonin on RRAGC-mutant B-cell lymphoma. This study provides a novel and precise targeted therapy for lymphoma patients with RRAGC mutations.

## Material and method

### Reagents

Antibodies against to phospho-S2448 mTOR (#2971), mTOR (#2972), phospho-T389 S6K1 (#9205), S6K1 (#9202), Raptor (#2114), RagC (#9480), β-actin (#8457), HRP-labeled anti-mouse (#7076) and anti-rabbit (#7074) secondary antibody, Protein A Agarose Beads (#9863), Rabbit IgG Isotype Control (#3900) were from Cell Signaling Technology (Danvers, MA, USA). Cardamonin (#C8249), FLAG M2 affinity gel (#F1804) and everolimus (#E-068) were purchased from Sigma-Aldrich and Merck KGaA (Darmstadt, Germany).

### Cell culture

Given the absence of FL cell lines, we resorted to commonly used B cell lymphoma cell lines (SUDHL-4 and OCI-Ly7) as previously described [11, 12]. HEK-293T cells (National Collection of Authenticated Cell Cultures, Shanghai, China) were cultured in high glucose DMEM media. SUDHL-4 (National Collection of Authenticated Cell Cultures, Shanghai, China) and OCI-Ly7 (Meisen Chinese Tissue Culture Collections, Zhejiang, China) cells were cultured in RPMI1640 media. The media was supplemented with 10% FBS, 2 mM glutamine, penicillin (100 IU/mL), and streptomycin (100 µg/mL). All cells were maintained in a humidified atmosphereat of 5% CO_2_ at 37 °C. Solutions and supplements for cell culture were purchased from Gibco (Grand Island, NY, USA).

### Cell viability assay

The Cell Counting Kit-8 (CCK-8) assay kit (#96,992, Sigma Aldrich; Merck KGaA) was used to determine the cell viability. Briefly, 5 × 10^3^ cells/well were seeded in 96-well plate. The cells were treated with cardamonin for 48 h. 10 µL of CCK-8 solution was added into each well of the plate and then incubated for 2 h in the incubator at 37 °C. The absorbance was measured at 450 nm.

### Cell lysis and western blotting

Cells were rinsed once with ice-cold PBS and immediately lysed by RIPA lysis buffer (#9806, Cell Signaling Technology), which contained 20 mM Tris-HCl (pH 7.5), 150 mM NaCl, 1 mM Na_2_EDTA, 1 mM EGTA, 1% NP-40, 1% sodium deoxycholate, 2.5 mM sodium pyrophosphate, 1 mM β-glycerophosphate, 1 mM Na_3_VO_4_, 1 µg/mL leupeptin and 1 × protease/phosphatase inhibitor cocktail (#5872, Cell Signaling Technology) on ice for 30 min. Cell lysates were centrifuged at 15, 000 rpm in a microcentrifuge at 4 °C for 15 min. 35 µg of protein solutions were loaded and separated in a 6-12% SDS-PAGE gel. The full-length of target protein was cut out according to the protein marker, and then the protein was transferred onto the polyvinylidene difluoride membrane. The membranes were blocked by 5% non-fat milk and incubated with corresponding primary antibodies overnight at 4 °C following incubation with HRP-conjugated secondary antibodies at room temperature for 1 h. Immunoreactive proteins were visualised by HRP-enhanced chemiluminescence reagents. The protein blots were imaged by X-ray film exposure.

### Cell lysis and immunoprecipitation

For immunoprecipitation, cells were washed once with ice-cold PBS and lysed on ice for 30 min in lysis buffer (#9803, Cell Signaling Technology) containing 20 mM Tris-HCl (pH 7.5), 150 mM NaCl, 1 mM Na_2_EDTA, 1 mM EGTA, 1% Triton, 2.5 mM sodium pyrophosphate, 1 mM β-glycerophosphate, 1 mM Na_3_VO_4_, 1 µg/mL leupeptin. 1 × protease/phosphatase inhibitor cocktail (#5872; Cell Signaling Technology) was added in the lysis buffer prior to use. Cell lysates were centrifuged at 15, 000 rpm for 15 min at 4 °C. For anti-RagC-immunoprecipitation, RagC antibody was added into the pre-cleared cell lysates (1:50) and incubated with rotation overnight at 4 °C. And then 30 µL of 50% slurry of protein A Agarose was added and further incubated for 1 h [25]. For anti-FLAG-immunoprecipitation, the FLAG-M2 affinity gel was washed three times with lysis buffer. 30 µL of 50% slurry of the affinity gel was then added into the pre-cleared cell lysates and incubated with rotation for 2 h at 4 °C as previously described [11]. Then the beads were washed three times by lysis buffer containing 500 mM NaCl. Immunoprecipitated proteins were denatured by the addition of 50 µL of sample buffer and boiled for 5 min. The proteins were separated by 10% SDS-PAGE, and detected by western blotting using standard procedures.

### Viral transduction

The lentiviruses encoding either wild type (WT) or Thr90Asn (T90N) mutant form of RRAGC (gene ID: 64121) were constructed in GV643 (pRRLSIN-cPPT-SFFV-MCS-3FLAG-E2A-EGFP-SV40-puromycin) vector (Genechem Co., Ltd., Shanghai, China). Virus infection was performed as previously described [12]. HEK-293T and SUDHL-4 cells were infected by spin-inoculation at 30ºC at 2, 600 rpm using 8 µg/mL of polybrene for 2 h before seeding into fresh medium. 48 h later, the media was changed to fresh media containing puromycin for selection. Stably transfected cells were tested for expression levels of RagC proteins using RagC- and FLAG-directed antibodies and western blotting.

### Knockdown of Raptor

Raptor was silenced using lentivirus-mediated transfection of shRNA as previously described [25]. The target sequence of RAPTOR is: 4145 sense, CCGGAGGGCCCTGCTACTCGCTTTTCTCGAGAAAAGCGAGTAGCAGGGCCCTTTTTTG; 4145 antisense, AATTCAAAAAAGGGCCCTGCTACTCGCTTTTCT CGAGAAAAGCGAGTAGCAGGGCCCT. The number indicated the nucleotide position in the transcripts (with position 1 set at the start codon) at which the 21 bp stem of the shRNA begins. shRNA sequence of RAPTOR were constructed in GV248 (hU6-MCS-Ubiquitin-EGFP-IRES-puromycin) vector (Genechem Co., Ltd., Shanghai, China). SUDHL-4 cells were infected with lentivirus containing media for 24 h in the presence of 8 µg/mL Polybrene. The transfected cells were selected by puromycin and the resistant cells were used for experiments. Knockdown was confirmed by Western blot analysis.

### Xenograft experiments

8-weeks old female SCID mice were purchased from Beijing HFK bioscience Co., Ltd and housed in the Laboratory of Fujian Institute of Food and Drug Quality Control under specific pathogen-free conditions. 2 × 10^7^ SUDHL-4 cells were collected and washed with PBS twice. 200 µL of cell suspension were injected subcutaneously into the right flank of the mice with Matrigel (#356,234, BD Biosciences) in a mixture 1:1. Tumor volume was measured 2 times per week with calipers and calculated using the following formula: tumor volume (mm^3^) = 1/2 × length × width^2^. When the volume of the tumor reached to 50 mm^3^, the mice were randomized into control group (0.5% sodium carboxymethyl cellulose) and cardamonin (15 mg/kg) treatment groups. Intragastric administration of cardamonin was preformed once per day for 30 days. Then the mice were sacrificed, and the tumor was isolated. All animal works were approved by the Ethics Committee of Fujian Maternity and Child Health Hospital (Ethical approval No.: 2022KYLLD03088).

### Statistical analysis

All data were expressed as the mean ± standard deviation (mean ± SD). Statistical analysis was performed using SPSS 21.0 statistical software. Difference between two groups was performed by t-test and differences of multiple groups were determined by one-way ANOVA followed by Tukey-Kramer test for post hoc comparisons. *P* < 0.05 was considered significant.

## Results

### Cardamonin inhibits the viability of SUDHL-4 and OCI-Ly7 cells

**Fig. 1:**
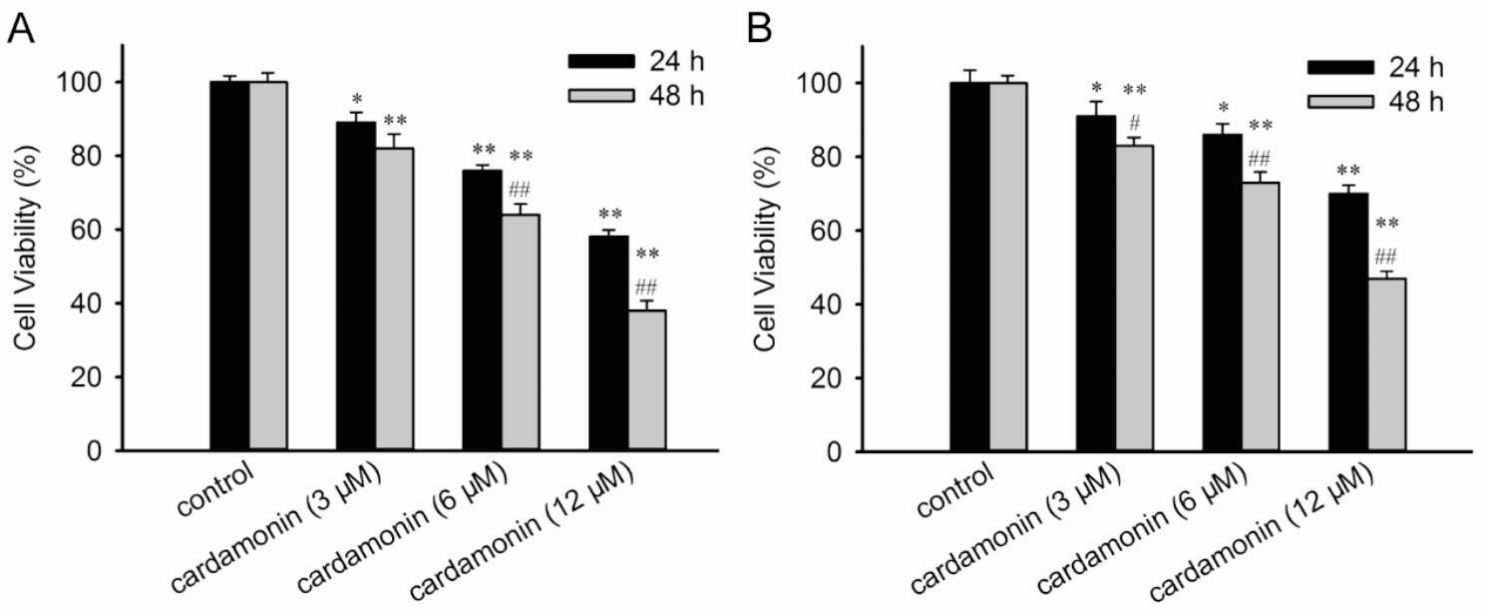
Viability of SUDHL-4 and OCI-Ly7 cells treated with inhibitory cardamonin. SUDHL-4 (**A**) and OCI-Ly7 (**B**) cells were treated with the indicated concentrations of cardamonin for 24 and 48 h. Cell viability was determined by CCK-8 assay. Data are presented as mean ± SD (*n = 3*). ^*^p < 0.05, ^**^p < 0.01 compared with the control; ^*##*^*p <* 0.01 compared at 24 h

To determine the therapeutic potency of cardamonin in lymphoma cells, its inhibitory effect on the viability of SUDHL-4 and OCI-Ly7 cells was measured using a CCK-8 assay. As shown in Fig. 1, treatment with increasing concentrations of cardamonin gradually decreased cell viability. The inhibitory effect of cardamonin on SUDHL-4 cell activity was greater than that on the activity of OCI-Ly7 cells.

### Cardamonin inhibits mTOR activation in SUDHL-4 and OCI-Ly7 cells

**Fig. 2:**
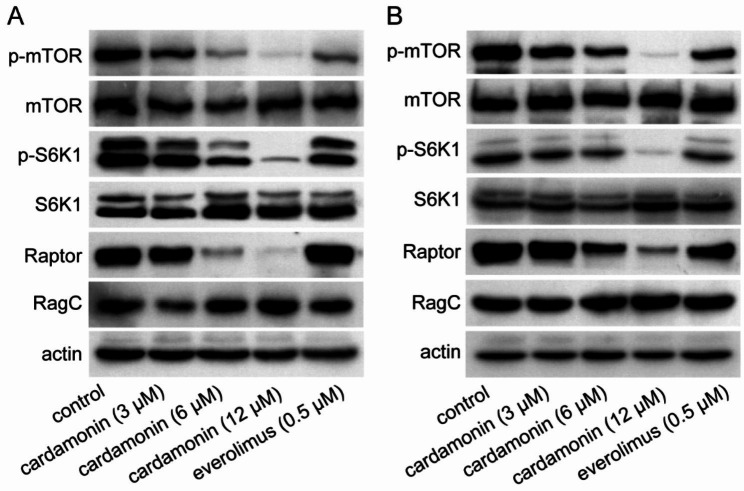
Cardamonin inhibits mTORC1 signalling and reduces the Raptor level. SUDHL-4 (**A**) and OCI-Ly7 (**B**) cells were treated with cardamonin at the indicated concentrations for 24 h. Phosphorylation rates of mTOR, mTOR signalling activity, and protein expression of mTORC1 components were analysed using immunoblotting (*n = 3*)

Next, the effect of cardamonin on mTORC1 signalling was determined. The phosphorylation of mTOR and its downstream substrate S6K1 was inhibited by cardamonin in both SUDHL-4 (Fig. 2A) and OCI-Ly7 cells (Fig. 2B). We measured the protein expression of mTORC1 components. As expected, the protein level of Raptor was decreased by cardamonin treatment, whereas that of RagC was unaffected. These results indicate that cardamonin specifically inhibits Raptor.

### Cardamonin disrupts the interaction of mTORC1 components with RagC

**Fig. 3:**
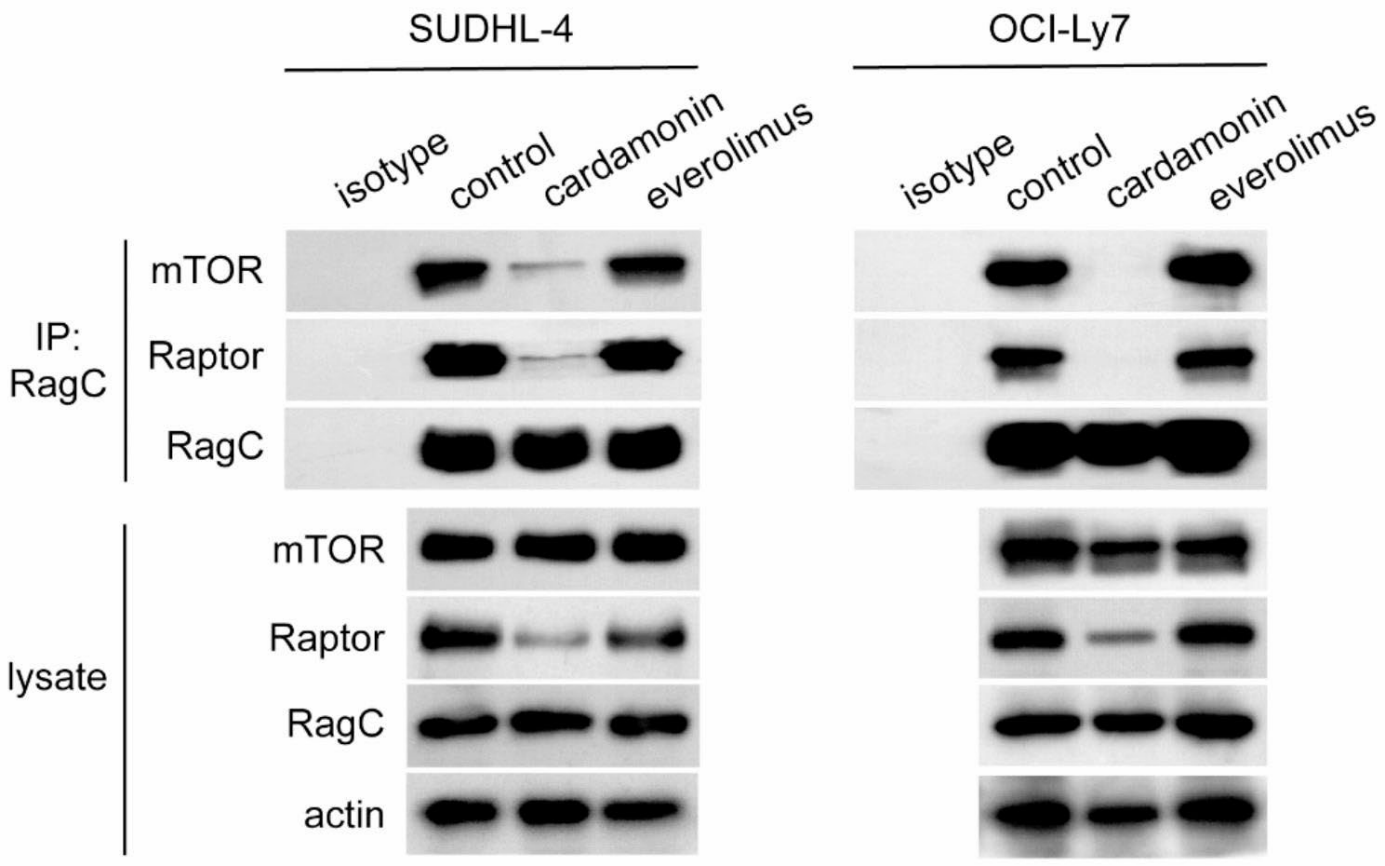
Cardamonin disrupts the interaction between RagC and mTOR. SUDHL-4 (**A**) and OCI-Ly7 cells (**B**) were treated with cardamonin (12 µM) or everolimus (0.5 µM) for 24 h. Immunoprecipitates pulled down by an anti-RagC antibody were collected. Cell lysates and immunoprecipitates were analysed by western blotting (*n = 3*)

Considering the important role of Raptor in mTOR activation, we further examined whether decreased Raptor expression affected the formation of the mTOR-activating complex. Cardamonin disrupted the interaction between Raptor and RagC as well as the connection between mTOR and RagC. Because everolimus inhibited mTOR in a manner different from that of cardamonin, the effect of everolimus on the formation of the mTOR-activating complex was further determined. As expected, everolimus did not affect the interaction between mTORC1 components (Fig. 3). This indicates that cardamonin may be a novel mTOR inhibitor.

### RRAGC-T90N mutation increases mTOR activation and cell proliferation

**Fig. 4:**
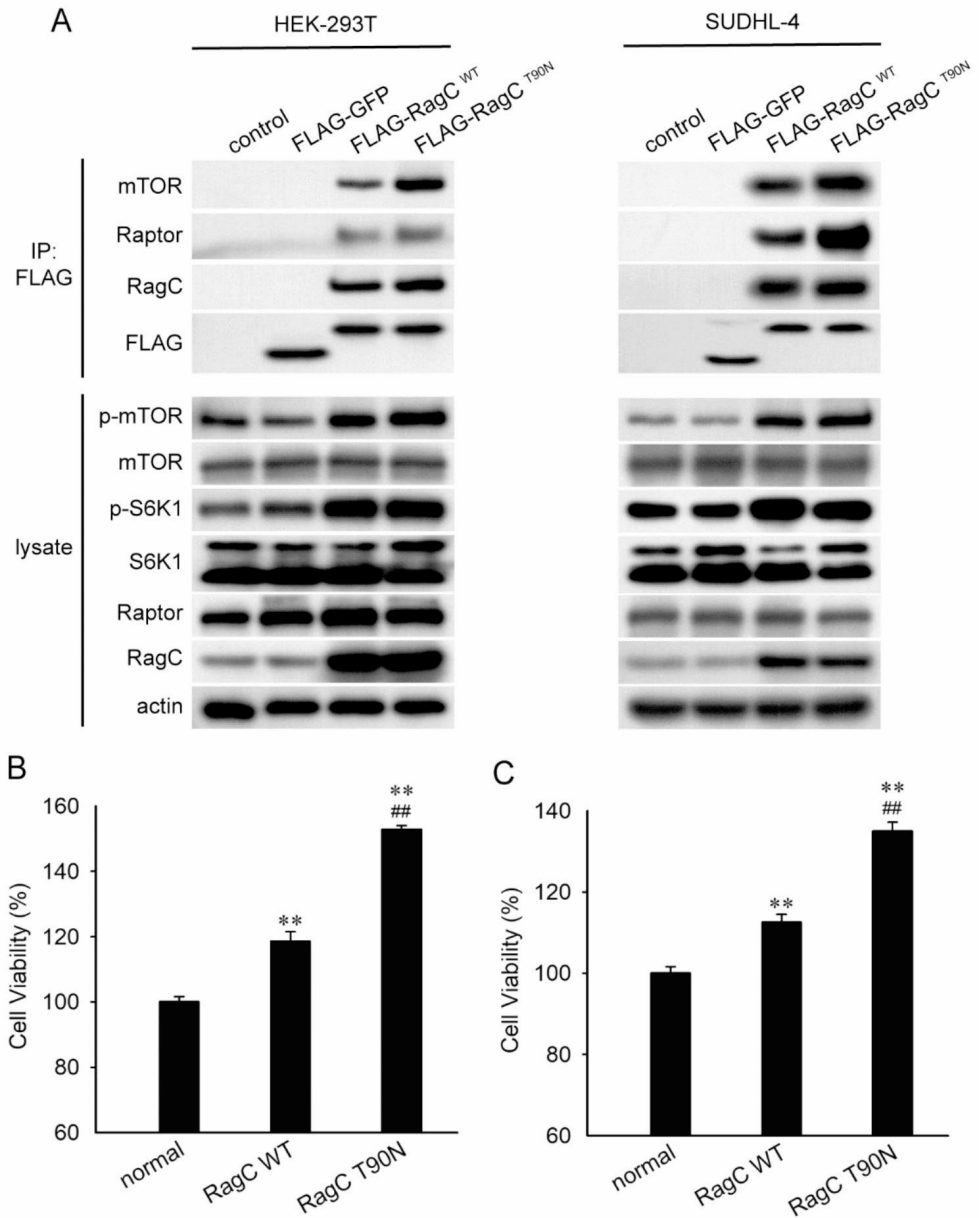
Effects of the RagC^T90N^ mutant on mTORC1 signalling. HEK-293T or SUDHL-4 cells were transfected with lentiviruses expressing FLAG-GFP, FLAG-RagC^WT^ and FLAG-RagC^T90N^. (**A**) Immunoprecipitates pulled down by an anti-FLAG antibody from cells expressing the indicated cDNAs were collected. Cell lysates and immunoprecipitates were analysed by western blotting (*n = 3*). (**B, C**) Cell viability of normal, FLAG-RagC^WT^- and FLAG-RagC^T90N^-expressing HEK-293T and SUDHL-4 cells was determined by CCK-8 assay. Data are presented as mean ± SD (*n = 3*). ^**^p < 0.01 compared with the normal group; ^*##*^*p <* 0.01 compared with the RagC^WT^ group

We employed stable lentivirus-transduced HEK-293T and SUDHL-4 cells expressing FLAG-RRAGC WT (RagC^WT^) and FLAG-RRAGC mutant T90N (RagC^T90N^). The binding capacity of RagC to Raptor was assessed by co-immunoprecipitation. As shown in Fig. 4A, the interaction between RagC and Raptor or mTOR increased, and RagC^T90N^ cells coimmunoprecipitated more Raptor than RagC^WT^ cells. Compared to normal cells, the phosphorylation of mTOR and S6K1 was increased in both RagC^WT^ and RagC^T90N^ cells. The hyperactivated mTOR signalling led us to speculate whether RagC^WT^ and RagC^T90N^ mutations confer a growth or proliferative advantage. The results of the CCK-8 assay showed that the cell viability of RagC^WT^ and RagC^T90N^-mutant HEK-293T and SUDHL-4 cells was increased by nearly 10–20% and 40–60%, respectively. Furthermore, the viability of RagC^T90N^-mutant cells was increased than that of RagC^WT^ cells (Fig. 4B, C).

### RRAGC mutation renders SUDHL-4 sensitive to cardamonin

**Fig. 5:**
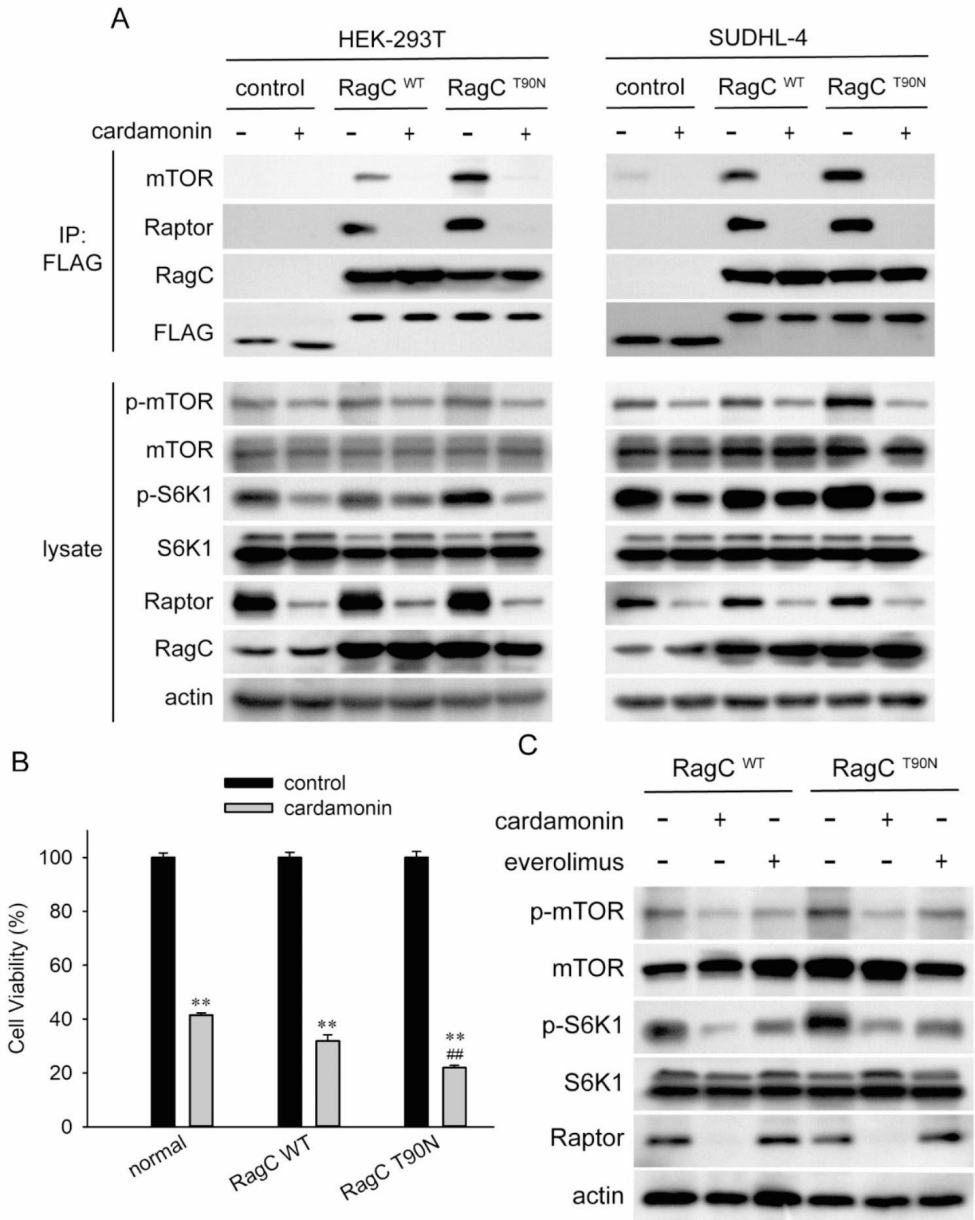
RRAGC mutation renders SUDHL-4 sensitive to cardamonin. Normal, RagC^WT^ and RagC^T90N^-mutant HEK-293T or SUDHL-4 cells were treated with cardamonin (12 µM). (**A**) Immunoprecipitates pulled down by an anti-FLAG antibody were collected from cells expressing the indicated cDNAs. Cell lysates and immunoprecipitates were analysed by western blotting (*n = 3*). (**B**) Cell viability of normal, RagC^WT^ and RagC^T90N^-mutant SUDHL-4 cells was determined by CCK-8 assay. Data are presented as mean ± SD (*n = 3*). ^**^p < 0.01 compared with the control group; ^##^*p* < 0.01 compared with the cardamonin-treated RagC^WT^ group. (**C**) RagC^WT^ and RagC^T90N^-mutant SUDHL-4 cells were treated with cardamonin (12 µM) or everolimus (0.5 µM). Cell lysates were analysed by western blotting to measure the level of the indicated proteins (*n = 3*)

RRAGC mutations enhance the affinity of RagC for Raptor, thereby inducing mTOR activation. These results demonstrated that cardamonin inhibited mTOR signalling by decreasing the protein level of Raptor. This finding prompted us to determine whether RRAGC-mutant cells were more sensitive to cardamonin. As expected, cardamonin exhibited a stronger inhibitory effect on the phosphorylation of mTOR and S6K1 in RagC^T90N^-mutant cells. Consistent with its strong inhibition on mTOR signalling, the interaction between RagC and Raptor or mTOR was significantly decreased by cardamonin (Fig. 5A). The viability of RagC^T90N^-mutant SUDHL-4 cells revealed that they were more sensitive to cardamonin than were normal or RagC^WT^ cells. The inhibitory effect of cardamonin on the viability of RagC^T90N^-mutant cells was stronger than that on RagC^WT^ cells (Fig. 5B). However, everolimus inhibited mTOR phosphorylation at the same rate in both cell lines (Fig. 5C).

### Raptor knockdown abolishes the inhibitory effect of cardamonin on mTOR

**Fig. 6:**
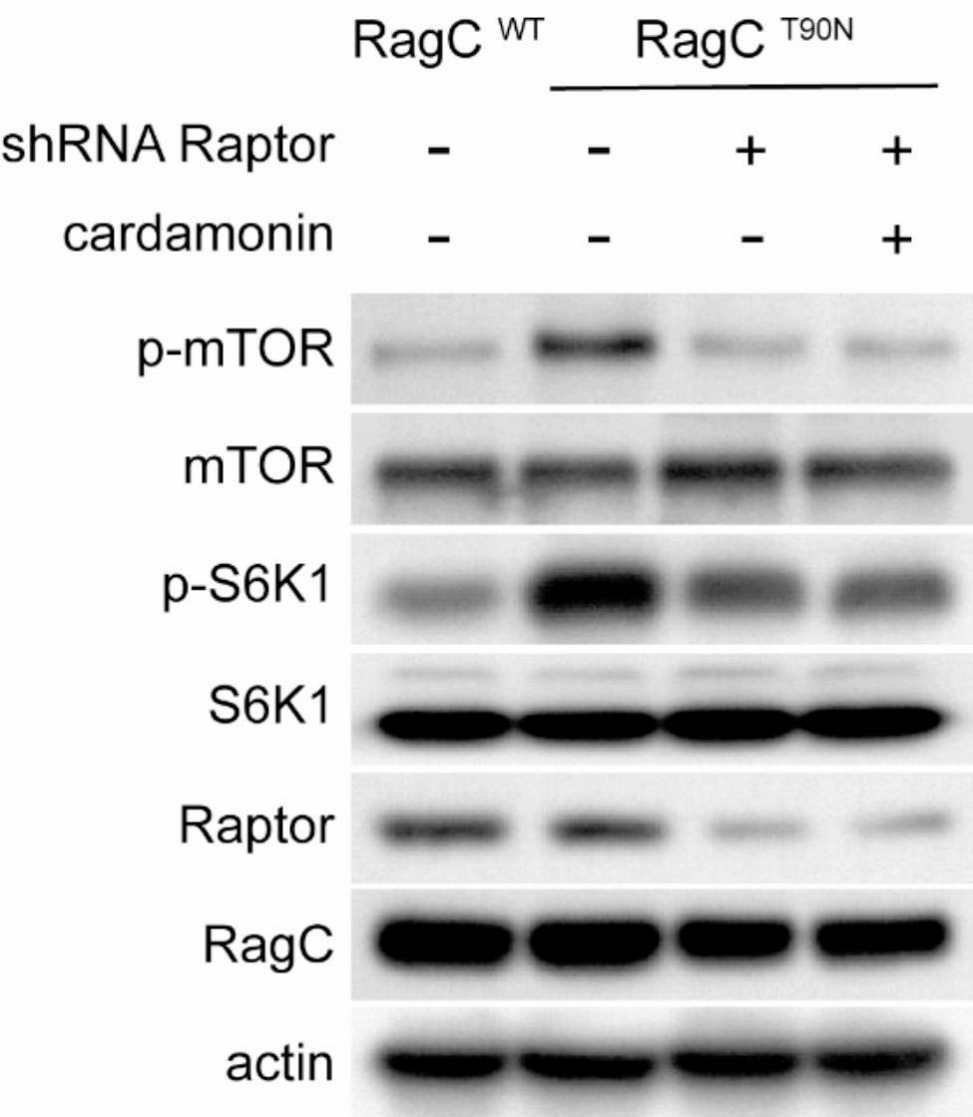
Raptor shRNA abolishes the inhibitory effect of cardamonin on mTOR signalling in RRAGC-mutant cells. Protein expression of Raptor was knocked down by shRNA in RagC^T90N^-mutant SUDHL-4 cells. Then, cells were treated with cardamonin (12 µM). Cell lysates were analysed by immunoblotting to measure the levels of the indicated proteins (*n = 3*)

To confirm whether Raptor mediates the inhibitory effect of cardamonin on mTOR, Raptor-knockdown cells were constructed using shRNA. As expected, Raptor shRNA decreased the phosphorylation of mTOR and S6K1. Furthermore, cardamonin exerted no additional inhibitory effects on mTOR in Raptor-knockdown cells (Fig. 6). Thus, cardamonin suppressed the activation of the mTOR signalling pathway, at least in part, by directly decreasing the protein level of Raptor.

### RRAGC mutation renders mice selectively sensitive to cardamonin

**Fig. 7:**
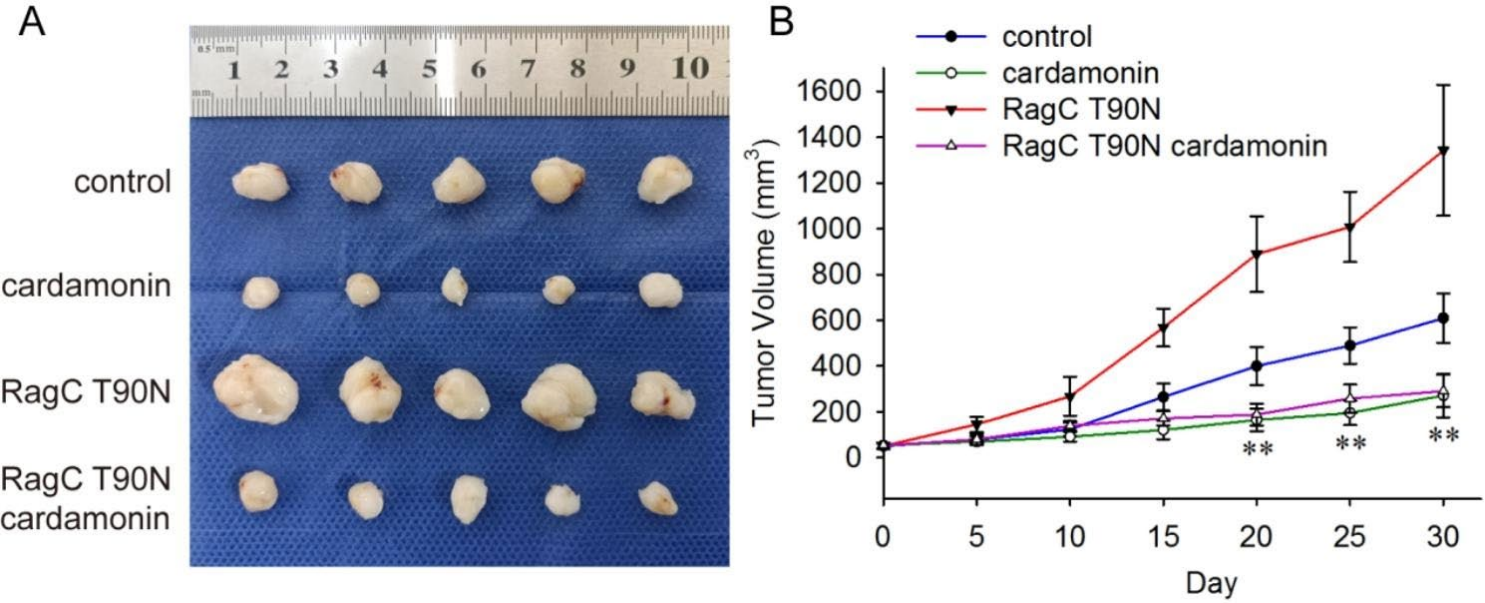
Cardamonin inhibits the growth of the RagC^T90N^-mutant SUDHL-4 in vivo. Mice bearing normal or RagC^T90N^ mutant xenograft tumours (*n* = 5 for each group) were randomly assigned to two different groups: (1) control and (2) cardamonin (15 mg/kg) group. Tumour size was measured with a calliper, and the tumour volume was calculated. Results are presented as the tumour volume (mm^3^) ± SD. ^**^p < 0.01 compared with the control or RagC^T90N^-mutant-bearing mice

Finally, we determined the inhibitory effects of cardamonin on RRAGC-mutant lymphomas in vivo. Mouse xenografts implanted with SUDHL-4 cells expressing mutant RRAGC exhibited a significantly higher tumour growth rate than those expressing WT RRAGC. Cardamonin inhibited the growth of RagC^WT^ and RagC^T90N^-mutant SUDHL-4 cells in vivo. In addition, xenografts implanted with the RagC^T90N^-mutant exhibited a significantly higher sensitivity to cardamonin treatment (Fig. 7).

## Discussion

Patients with FL usually experience a relapse-remitting disease course. Although chemotherapy is an effective treatment for FL, the cumulative toxicity of the chemotherapeutic agents can lead to early mortality. The availability of anti-CD20-based therapies has provided sufficient evidence to show that targeted agents can prolong the 5-year overall survival of patients with FL. In recent years, increasing evidence has demonstrated that targeted drugs, including tyrosine kinase inhibitors, mTOR inhibitors, PI3-kinase inhibitors, and enhancer of zeste homolog 2 inhibitors, exhibit attractive therapeutic efficacy against FL [26].

mTOR is an important regulator of many fundamental cellular processes including metabolism, proliferation and survival [5]. Activation of mTOR depends on the nucleotide-binding state of Rag GTPases. The “active” RagA/B·GTP-RagC/D·GDP heterodimer binds directly to Raptor following the activation of mTOR [13]. The core structure of RagC consists of a C-terminal CRD and an N-terminal GTPase domain. The GTPase domain contains the Switch I region. The T90N mutation causes a disorder in switch I, which results in decreased affinity for GTP and a preference for GDP [11, 13]. In accordance with the increased binding capacity of RRAGC-mutant cells to GDP, these results demonstrated that RRAGC-mutant-expressing cells recruited more Raptor than wild-type RagC-expressing cells. Furthermore, the phosphorylation of mTOR signalling and cell viability increased in RRAGC-mutant cells, as well as the tumour growth rate of RRAGC-mutant-harbouring animals. Hence, oncogenic RRAGC mutation strongly enhances mTOR activation and accelerates B cell lymphoma cell proliferation. However, a potential therapeutic approach against Rag GTPase is yet to be developed, and its efficacy and safety remain unknown [27]. As RRAGC mutation results in hyperactivation of mTOR, targeting mTOR could be a therapeutic strategy for RRAGC-mutant lymphoma.

Rapalogs have been used in clinical trials for the treatment of various cancers, including lymphoma [28]. Temsirolimus exhibited significant anti-tumour activity in DLBCL and FL patients, and the duration of response and progression-free survival were longer in FL patients [29]. Everolimus was effective in patients with relapsed and/or refractory indolent NHL and in those with relapsed or refractory classical Hodgkin lymphoma [16, 30, 31]. However, some patients have shown impressive responses to rapalogs in clinical trials. A better understanding of the genetic mechanisms underlying FL development will improve patients’ outcomes [32]. Patients with FL harbour RRAGC mutations that specifically activate mTOR and accelerate lymphomagenesis. Therefore, the mutant cells were more sensitive to mTORC1 inhibitors [15]. Since cardamonin exerted an inhibitory effect on mTOR, we speculated that RRAGC-mutant lymphoma cells were selectively sensitive to cardamonin. As expected, the results demonstrated that cardamonin inhibited the activation of mTOR signalling and cell viability in RRAGC-mutant cells. In addition, the inhibitory effect of cardamonin on RagC ^T90N^-mutant cells was stronger than that on RagC^WT^ cells. These results confirmed that mTOR inhibitors exhibit selective affinity for RRAGC-mutant lymphomas. Recently, the combination of rapalogs with tyrosine kinase or histone deacetylase inhibitors has led to significant progress in the treatment of patients with relapsed refractory Hodgkin lymphoma [33, 34]. Based on these results, the anti-tumour effects of cardamonin alone and in combination with other agents need to be further evaluated in animal models of FL [35].

Rapalogs first form a complex with FKBP12, which then binds to the FKBP12/rapamycin-binding domain to block mTOR function [36, 37]. Our previous studies showed that cardamonin suppressed mTOR activation of by decreasing the protein level of Raptor [24, 38]. Recent studies demonstrated that cardamonin splits Raptor into smaller molecular species by activating caspases [39]. In addition, cardamonin inhibited the proliferation and phosphorylation of mTOR and S6K1 in rapamycin-resistant cells [40]. Therefore, cardamonin inhibited mTOR in a manner different from that of rapalogs. In this study, we found that the activation of mTOR signalling in RRAGC-mutant cells was more sensitive to cardamonin than to rapalogs. The cardamonin-induced decrease in Raptor expression further disrupted the formation of the mTOR-activating complex and led to a more efficient inhibition of RRAGC-mutant lymphomas. We speculated that RRAGC-mutant-induced mTOR activation makes Raptor an efficient therapeutic target.

In addition to FL, a *de novo* mTORC1-activating RRAGC mutation (RagC^S75Y^) has been found in patients with dilated cardiomyopathy [41, 42]. Overexpression of the RRAGC^S75Y^-mutant in neonatal rat ventricular cardiomyocytes led to hyperactive mTORC1 signalling [43]. In contrast to its effects on lymphoma cells, mTOR inhibition did not ameliorate the acquisition of cardiac phenotypes in RRAGC-mutant cardiomyopathy. Rag GTPases mediate the translocation of transcription factor EB (TFEB) to lysosomes, where it is further phosphorylated by mTORC1. Once phosphorylated, nuclear translocation of TFEB is limited, triggering a cascade of pathological changes, such as cardiomyopathy [43, 44]. Recent studies have demonstrated that mTORC1-mediated TFEB phosphorylation in lysosomes is regulated by RagD, but is affected to a lesser extent by RagC [45]. Therefore, mTOR inhibition failed to restore nuclear translocation of TFEB or ameliorate cardiomyopathy in RRAGC-mutant-related dilated cardiomyopathy. However, in the present study, we did not analyse the translocation or phosphorylation of TFEB in the lysosomes. It remains to be determined whether TFEB phosphorylation occurs in lymphocytes and whether TFEB affects their susceptibility to mTOR inhibition.

However, this study had some limitations. First, the results do not directly reflect the inhibitory effect of cardamonin on RRAGC-mutant FL because of the absence of FL cell lines and their replacement with DLBCL cells in our experiments. Second, in the Raptor shRNA experiment, we concluded that Raptor partially mediated the inhibitory effect of cardamonin on RRAGC-mutant B-cell lymphoma. However, we did not consider the effects of Raptor overexpression. In another study, we detected the inhibitory effect of cardamonin on both Raptor knockdown and overexpression cell (unpublished data). Raptor overexpression in Raptor knockdown-cells restores mTOR signaling. As expected, cardamonin inhibited Raptor overexpression-induced mTOR activation. Based on these results, we speculate that Raptor mediates the inhibitory effect of cardamonin on mTOR. Next, we evaluated the inhibitory effects of cardamonin on cell viability. However, it is unknown whether cardamonin inhibits cell proliferation, induces apoptotic cell death, or disturbs the cell cycle. This issue needs to be investigated in future studies.

## Conclusions

**Fig. 8:**
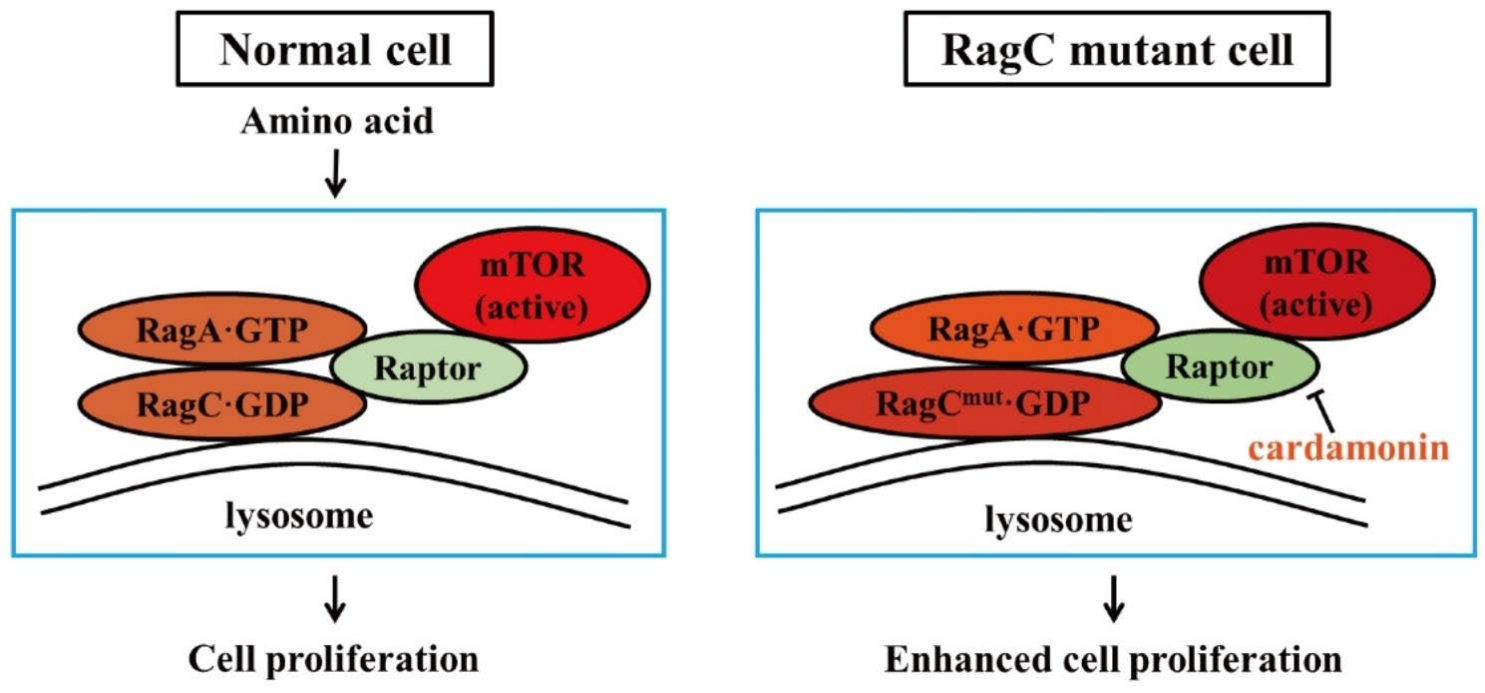
Inhibition mechanism of cardamonin on the RagC^T90N^-mutant cell. (Left) In normal cells, the “active” RagA·GTP-RagC·GDP heterodimer binds with Raptor, following the activation of mTOR in the presence of amino acids. (Right) RagC mutation increases its binding capacity with Raptor, which ultimately elicits hyperactivated mTORC1 signalling and enhancing the cell proliferation of lymphoma cells. Cardamonin decreases the protein level of Raptor and further disrupts the formation of mTOR activating complex. Pharmacological inhibition of mTORC1 by cardamonin exerts selective therapeutic effects on the RagC^T90N^-mutant lymphoma cells

The results of the present study led us to speculate whether the RRAGC mutation is a potential biomarker for the precise treatment of FL. Herein, we report that pharmacological inhibition of mTORC1 by cardamonin exerts selective therapeutic effects on RagC^T90N^-mutant lymphomas (Fig. 8). The present data justify future efforts to develop cardamonin as an individualised therapeutic drug for patients with FL harbouring RRAGC mutations.

### Electronic supplementary material

Below is the link to the electronic supplementary material.

Supplementary Material 1

Supplementary Material 2

Supplementary Material 3

Supplementary Material 4

Supplementary Material 5

Supplementary Material 6

## Data Availability

This document contains most of the data. Upon reasonable request, Peiguang Niu, the author of this manuscript, will provide more information. Email: npg4031@163.com.

## Abbreviations

CCK-8: Cell Counting Kit-8
FL: Follicular lymphoma
FKBP12: FK506-binding protein 12
GDP: guanosine diphosphate
GTP: guanosine triphosphate
mTORC1: mechanistic target of rapamycin complex 1
NHL: non-Hodgkin lymphoma
Raptor: regulatory protein associated with mTOR
Rag GTPase: Rag guanosine triphosphatase
RRAGC: Rag GTPase protein C
S6K1: p70 S6 kinase 1
