# Supplementary material for: Raptor mediates the selective inhibitory effect of cardamonin on RRAGC-mutant B cell lymphoma
Source: BMC Complement Med Ther. 2023 Sep 26;23:336. doi: 10.1186/s12906-023-04166-7 (PMC10521446; doi:10.1186/s12906-023-04166-7)
Supplement: Supplementary file 1 — Supplementary Material 1 [file 12906_2023_4166_MOESM1_ESM.docx]

Supplementary Original western blot images for Figure 2. Original western blotting for mTOR signalling, Raptor and RagC of the cardamonin and everolimus treated (A) SUDHL-4 and (B) OCI-Ly7 cells. The protein blots are imaged by X-ray film exposure.

Figure 2


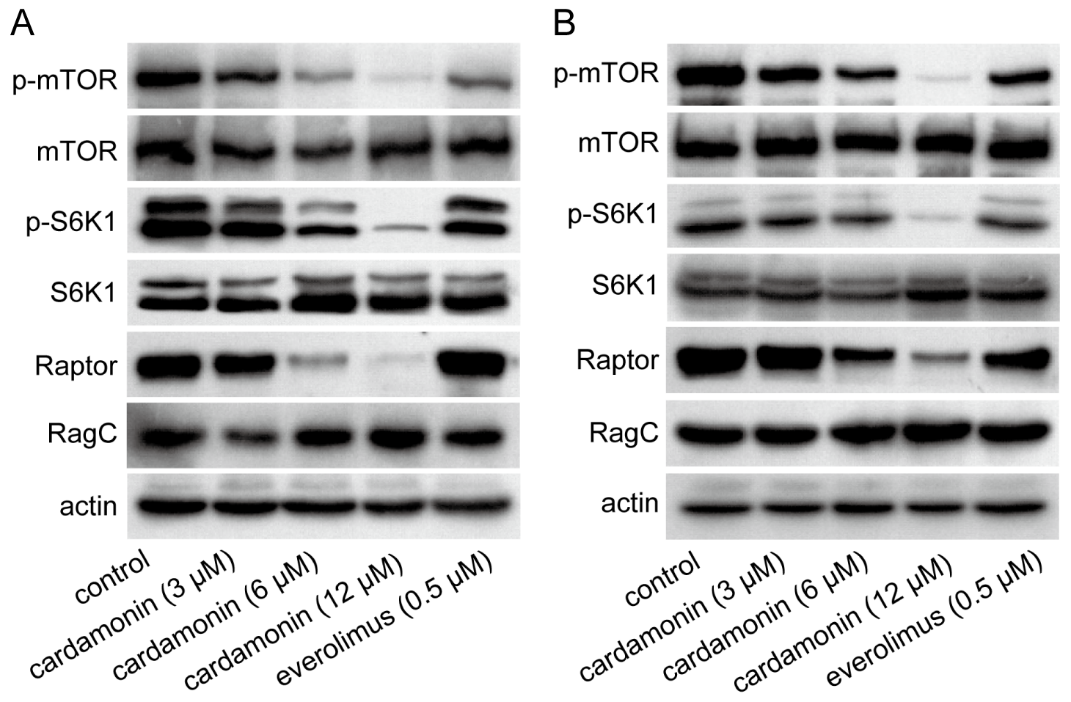



 Fig.2A SUDHL-4 p-mTOR


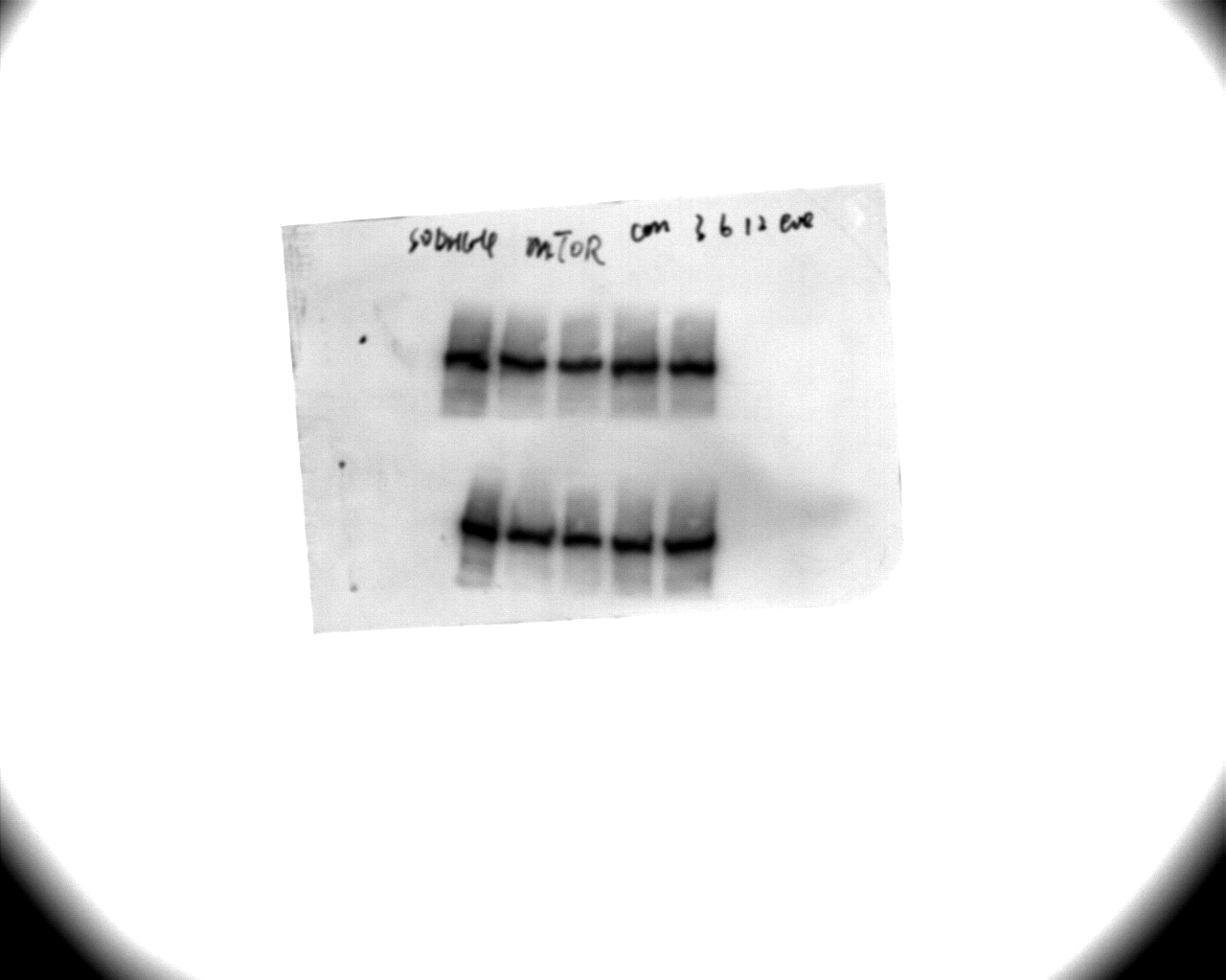
 Fig.2A SUDHL-4 mTOR



 Fig.2A SUDHL-4 p-S6K1


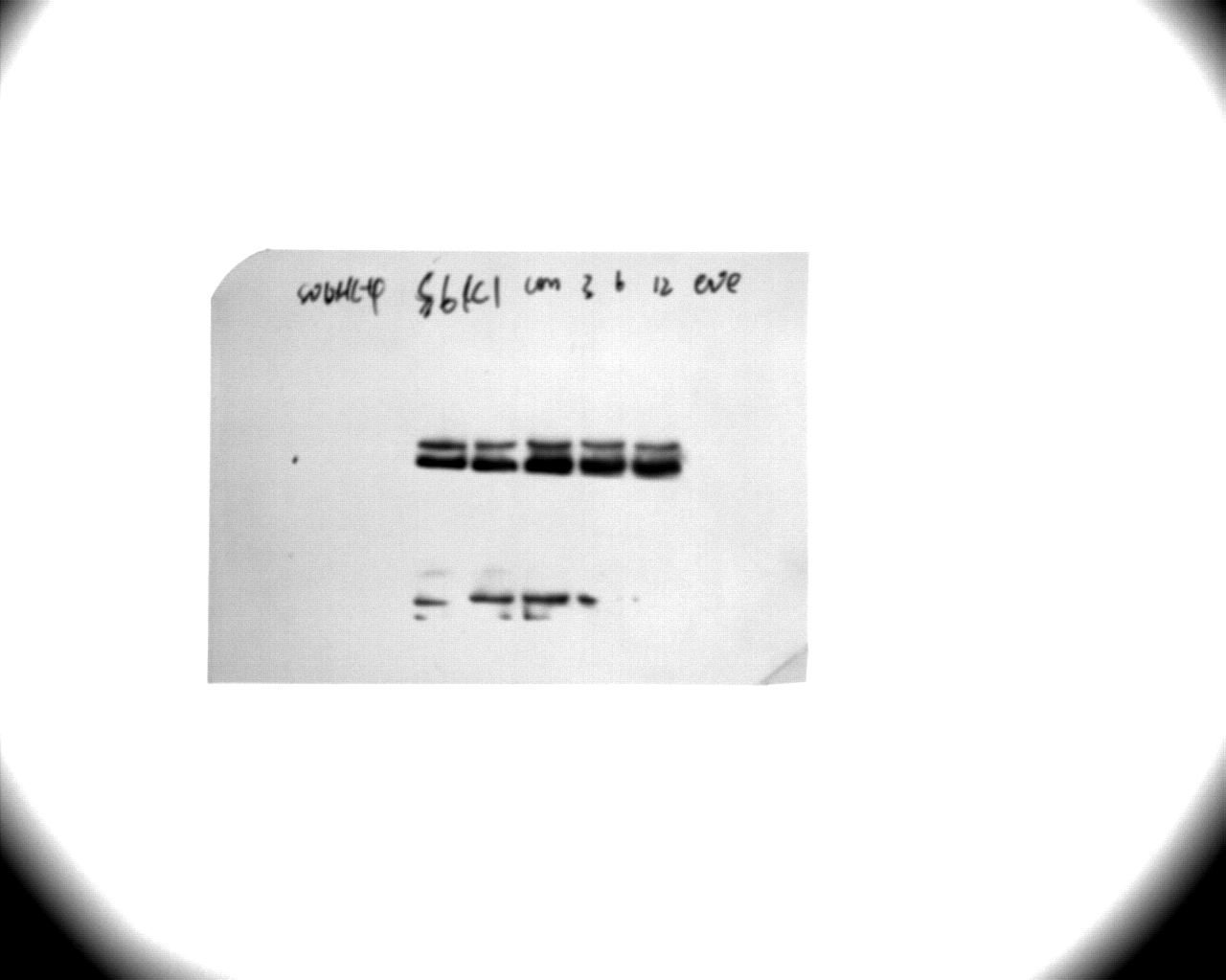


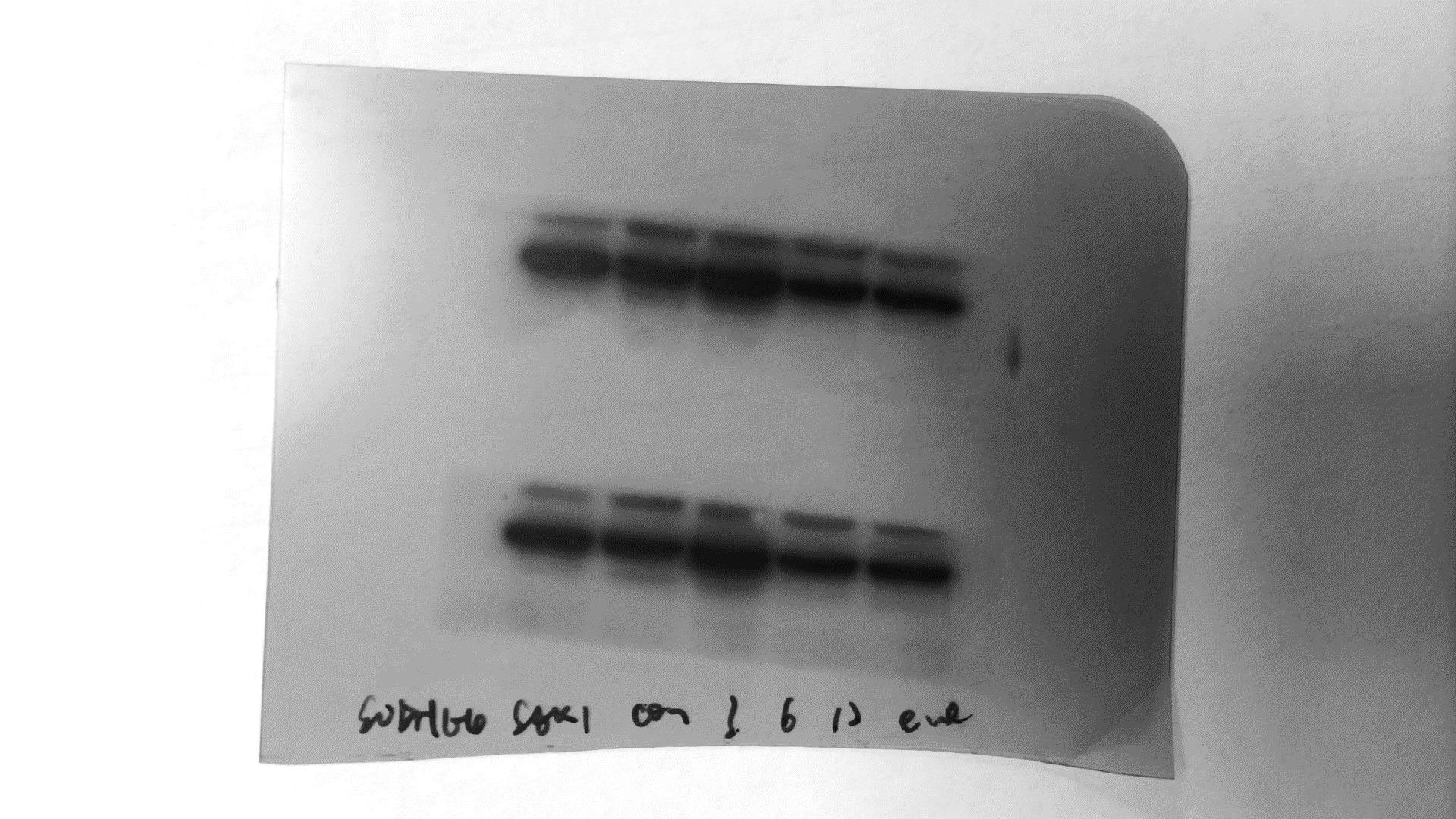
Fig.2A SUDHL-4 S6K1



 Fig.2A SUDHL-4 Raptor


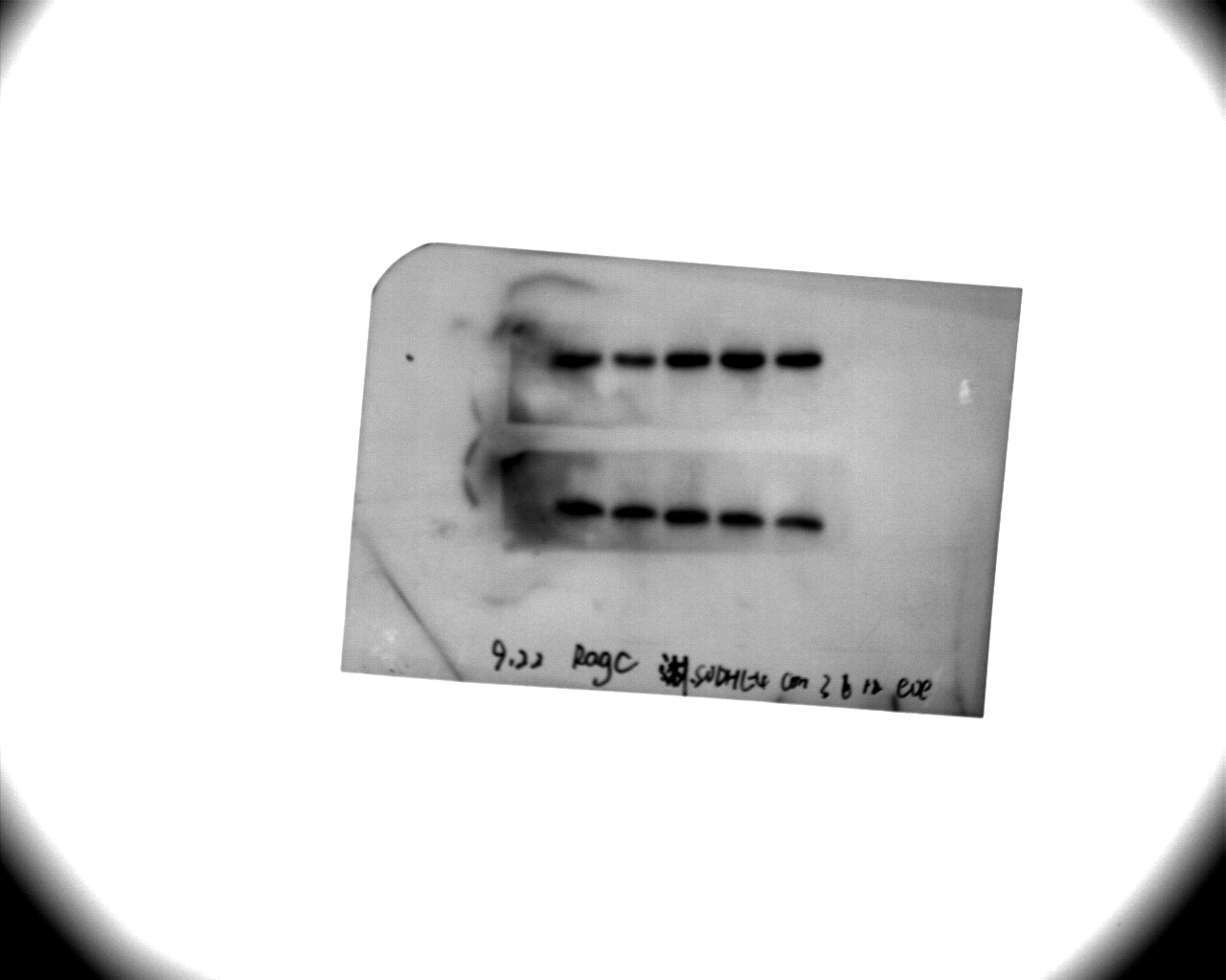
 Fig.2A SUDHL-4 RagC


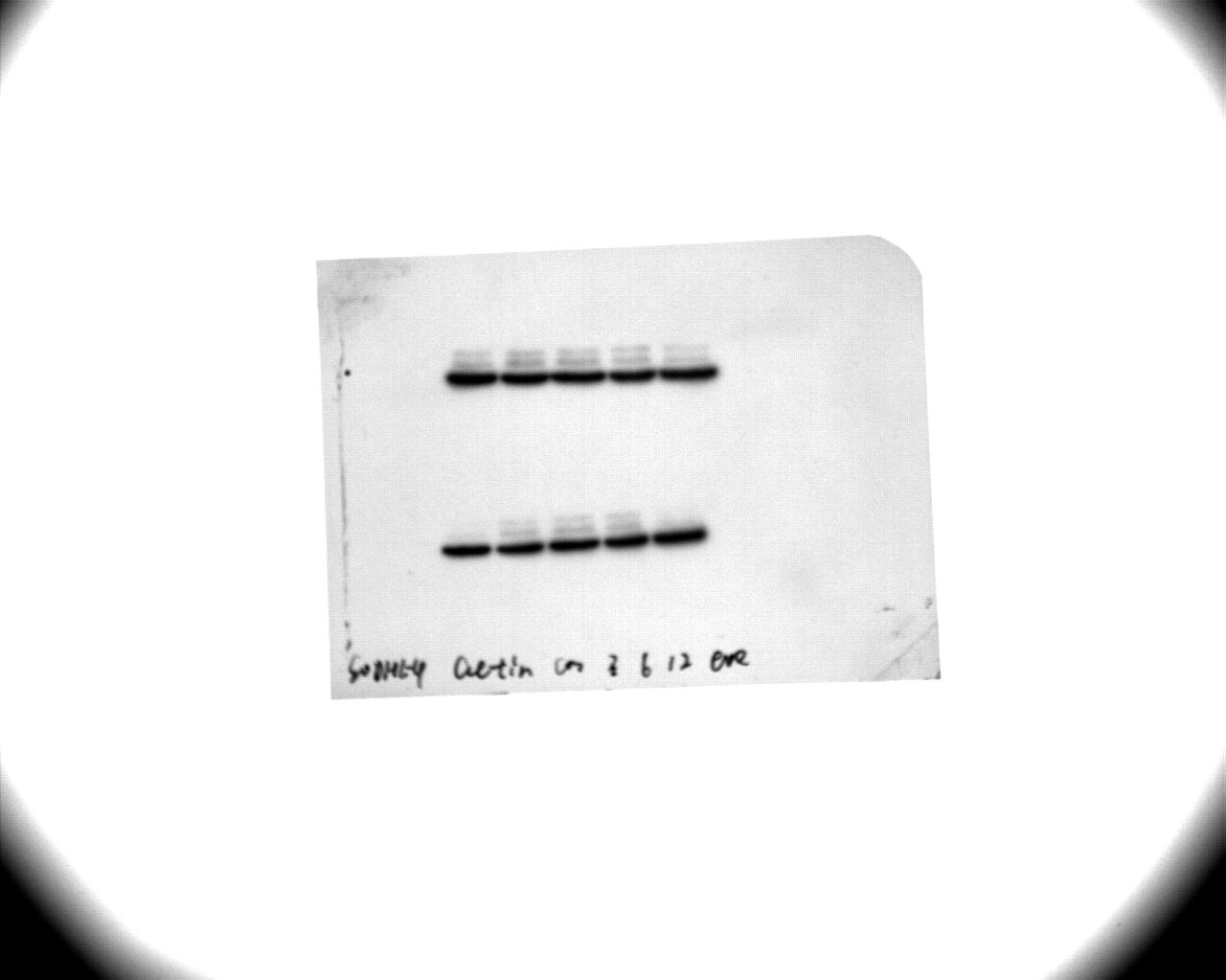
 Fig.2A SUDHL-4 actin


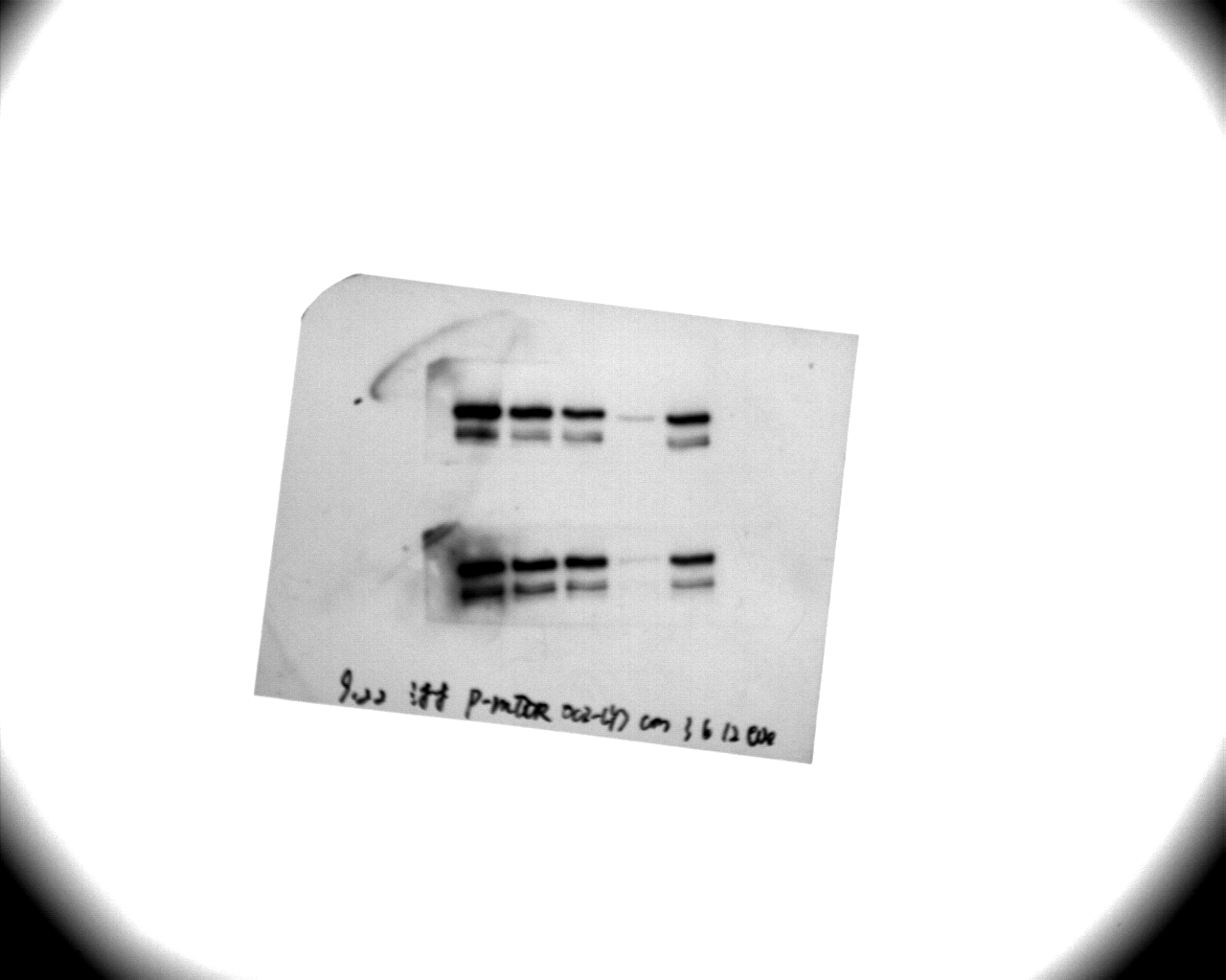
 Fig.2B OCI-Ly7 p-mTOR


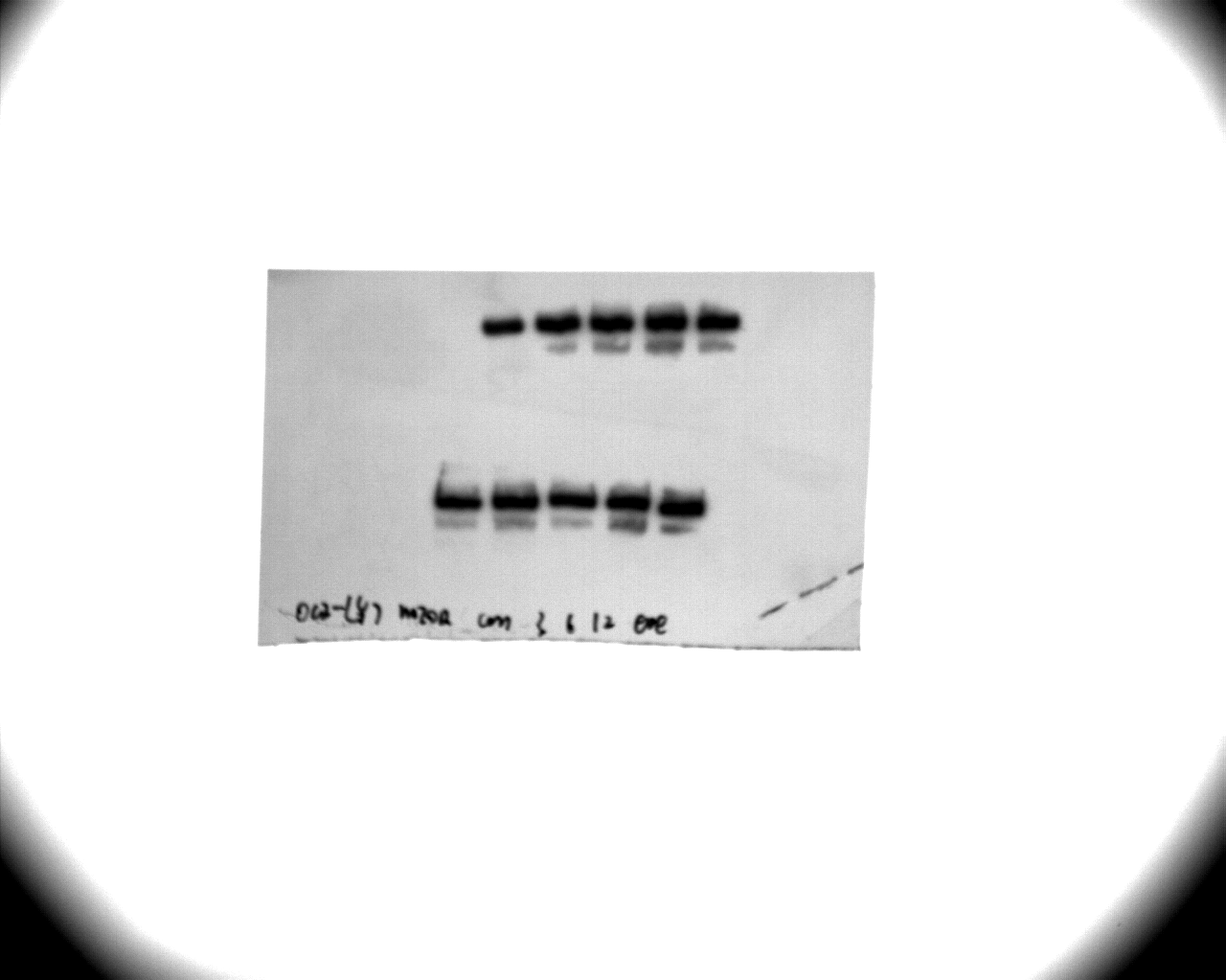
 Fig.2B OCI-Ly7 mTOR



 Fig.2B OCI-Ly7 p-S6K1


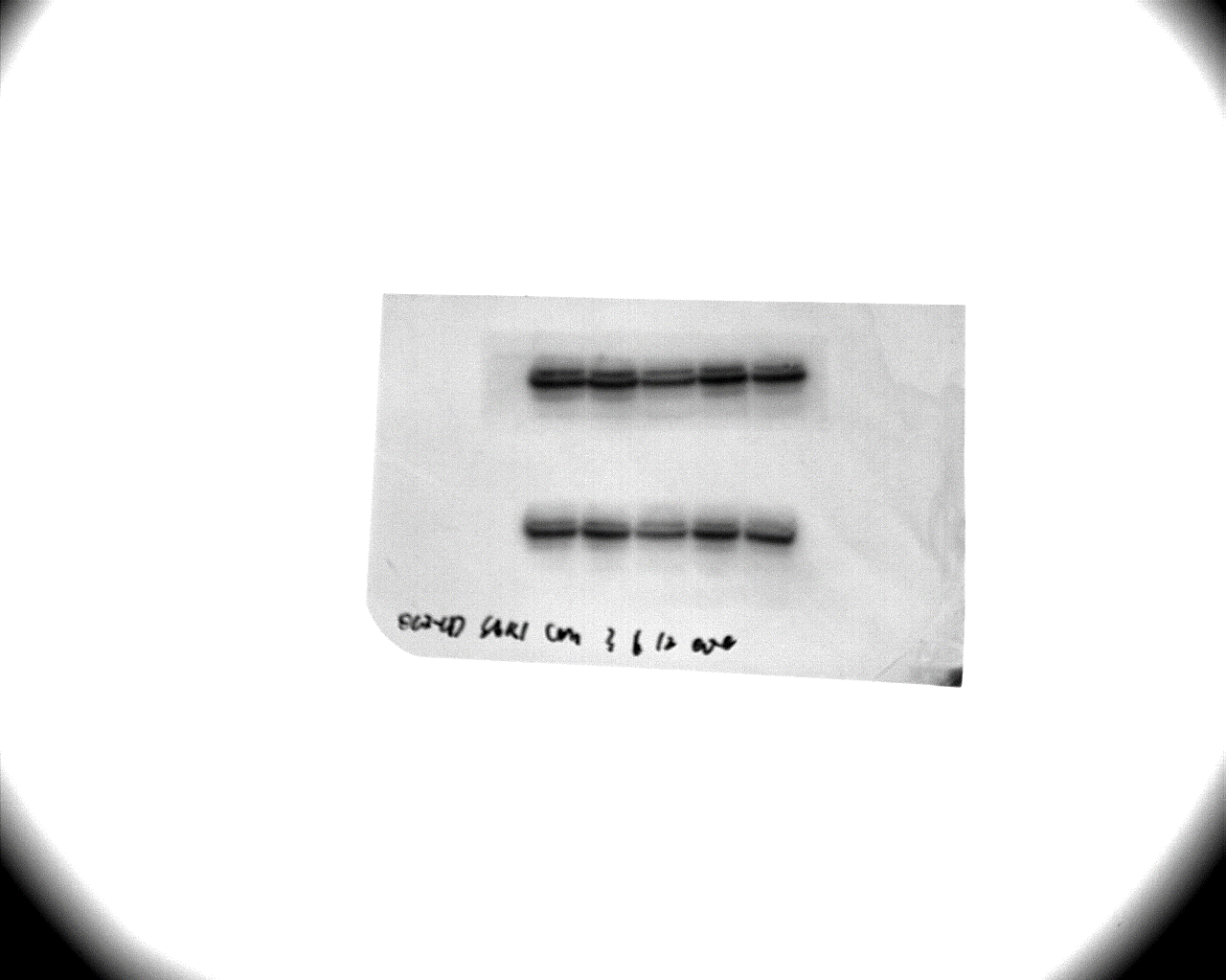
 Fig.2B OCI-Ly7 S6K1


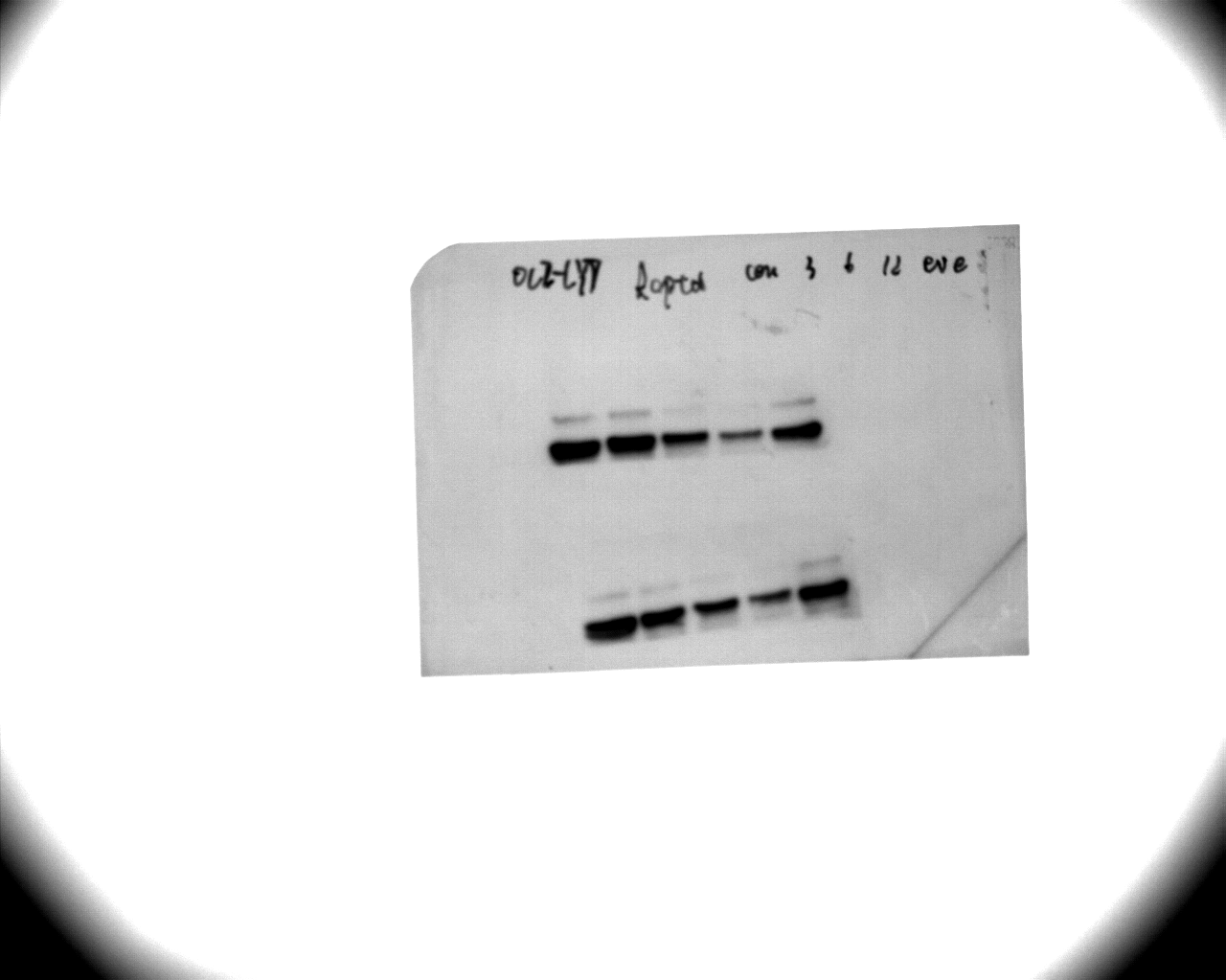
 Fig.2B OCI-Ly7 Raptor


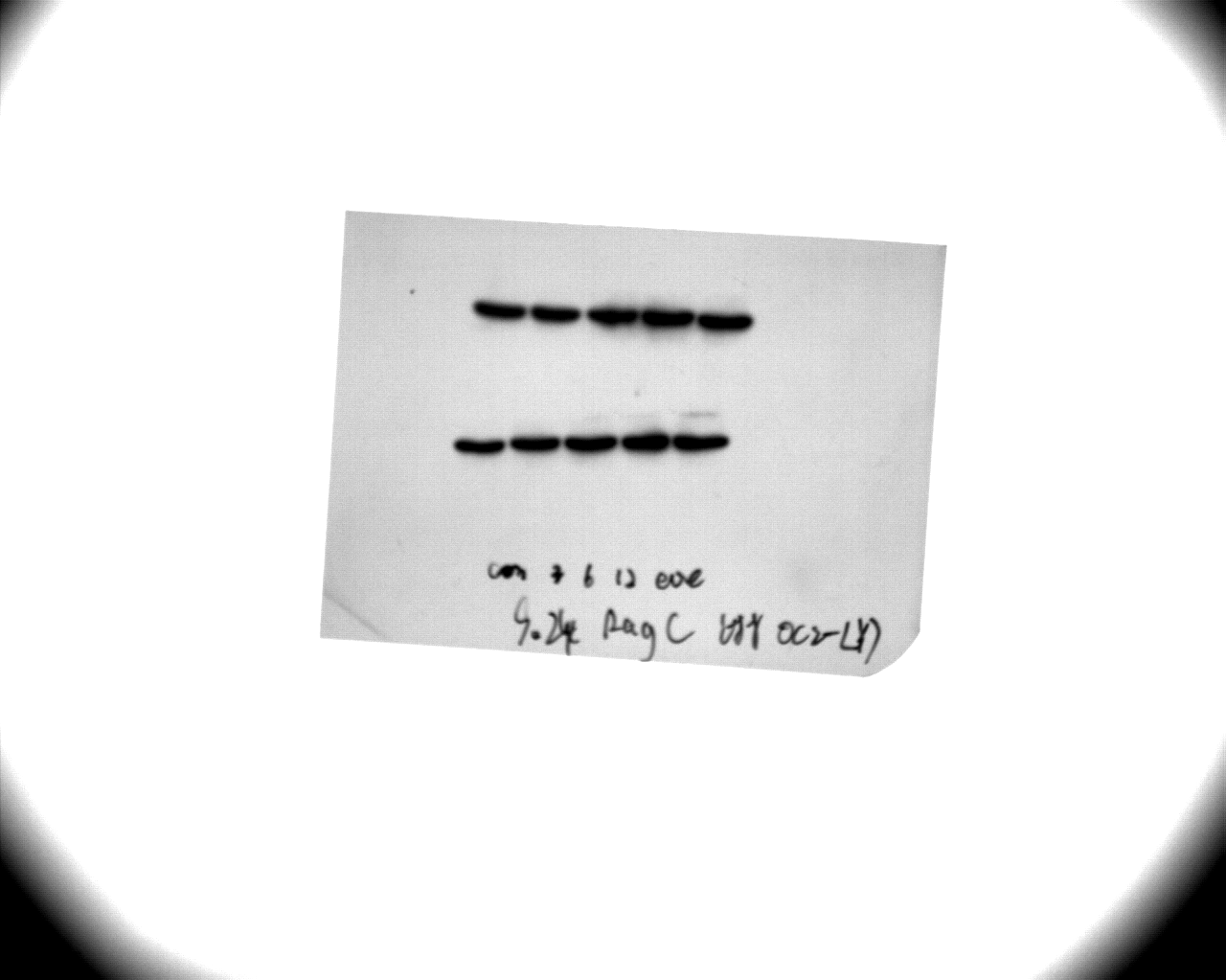
 Fig.2B OCI-Ly7 RagC






Fig.2B OCI-Ly7 actin
