# Supplementary material for: Raptor mediates the selective inhibitory effect of cardamonin on RRAGC-mutant B cell lymphoma
Source: BMC Complement Med Ther. 2023 Sep 26;23:336. doi: 10.1186/s12906-023-04166-7 (PMC10521446; doi:10.1186/s12906-023-04166-7)
Supplement: Supplementary file 2 — Supplementary Material 2 [file 12906_2023_4166_MOESM2_ESM.docx]

Supplementary Original western blot images for Figure 3. Original western blotting for mTOR, Raptor and RagC of the cardamonin and everolimus treated SUDHL-4 and OCI-Ly7 cells. The protein blots are imaged by X-ray film exposure.

Figure 3


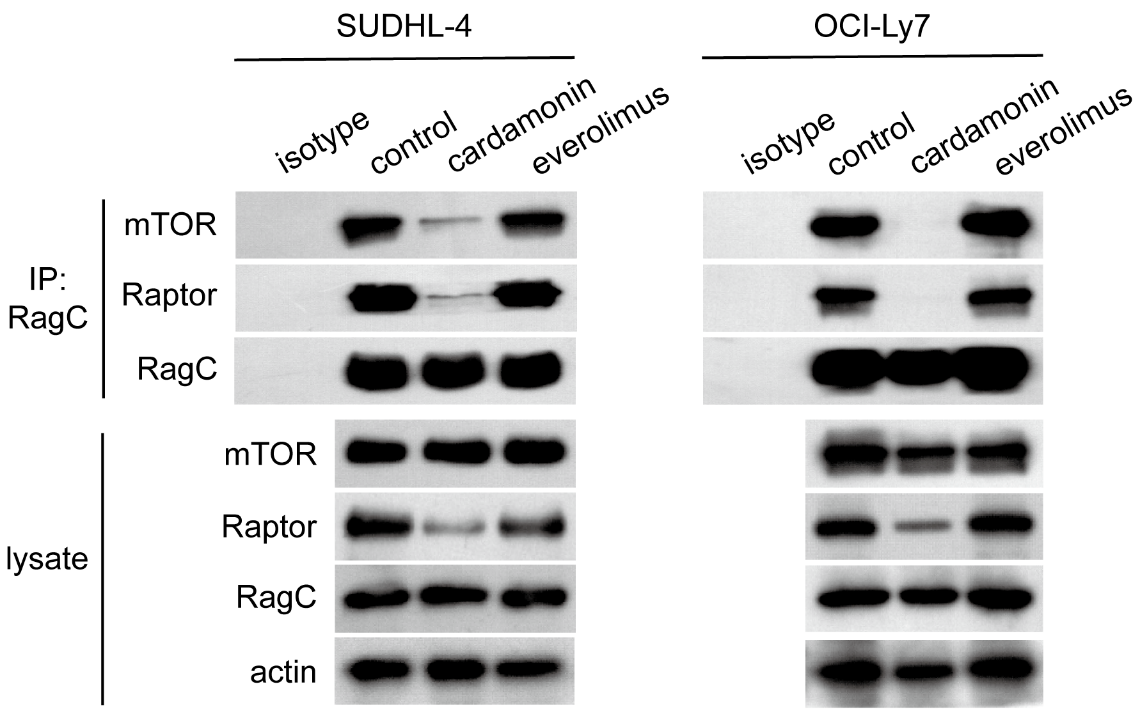



 Fig.3 SUDHL-4 IP mTOR



 Fig.3 SUDHL-4 IP Raptor



 Fig.3 SUDHL-4 IP RagC



 Fig.3 SUDHL-4 lysate mTOR



 Fig.3 SUDHL-4 lysate Raptor



Fig.3 SUDHL-4 lysate RagC



 Fig.3 SUDHL-4 lysate actin



 Fig.3 OCI-Ly7 IP mTOR



 Fig.3 OCI-Ly7 IP Raptor



 Fig.3 OCI-Ly7 IP RagC



 Fig.3 OCI-Ly7 lysate mTOR



Fig.3 OCI-Ly7 lysate Raptor



 Fig.3 OCI-Ly7 lysate RagC



 Fig.3 OCI-Ly7 lysate actin
