# Supplementary material for: Raptor mediates the selective inhibitory effect of cardamonin on RRAGC-mutant B cell lymphoma
Source: BMC Complement Med Ther. 2023 Sep 26;23:336. doi: 10.1186/s12906-023-04166-7 (PMC10521446; doi:10.1186/s12906-023-04166-7)
Supplement: Supplementary file 4 — Supplementary Material 4 [file 12906_2023_4166_MOESM4_ESM.docx]

Supplementary Original western blot images for Figure 5A. Original western blotting for mTOR signalling, Raptor and RagC of the FLAG-GFP, FLAG-RagC^WT^ and FLAG-RagC^T90N^ transfected HEK-293T and SUDHL-4 cells in the absent or present of cardamonin. The protein blots are imaged by X-ray film exposure.

Figure. 5A


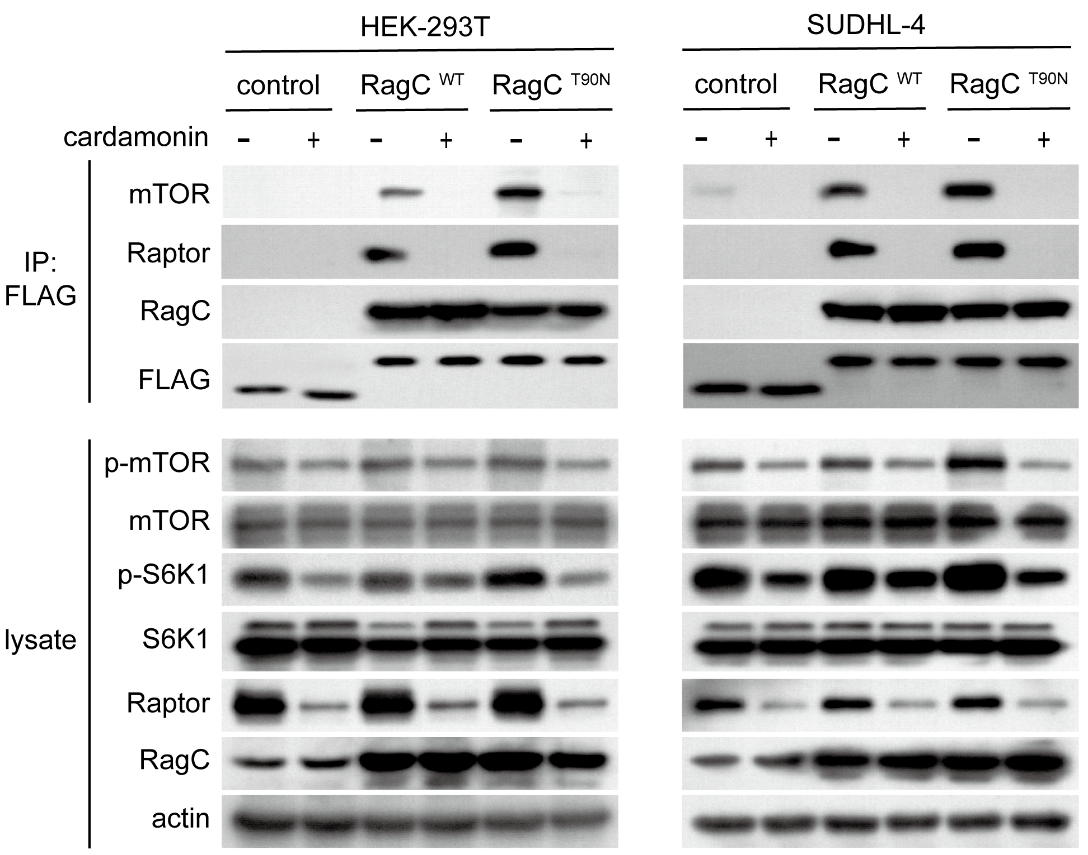



 Fig. 5A 293T IP mTOR



 Fig. 5A 293T IP Raptor



 Fig. 5A 293T IP RagC



 Fig. 5A 293T IP FLAG



 Fig. 5A 293T lysate p-mTOR



 Fig. 5A 293T lysate mTOR



 Fig. 5A 293T lysate p-S6K1



 Fig. 5A 293T lysate S6K1


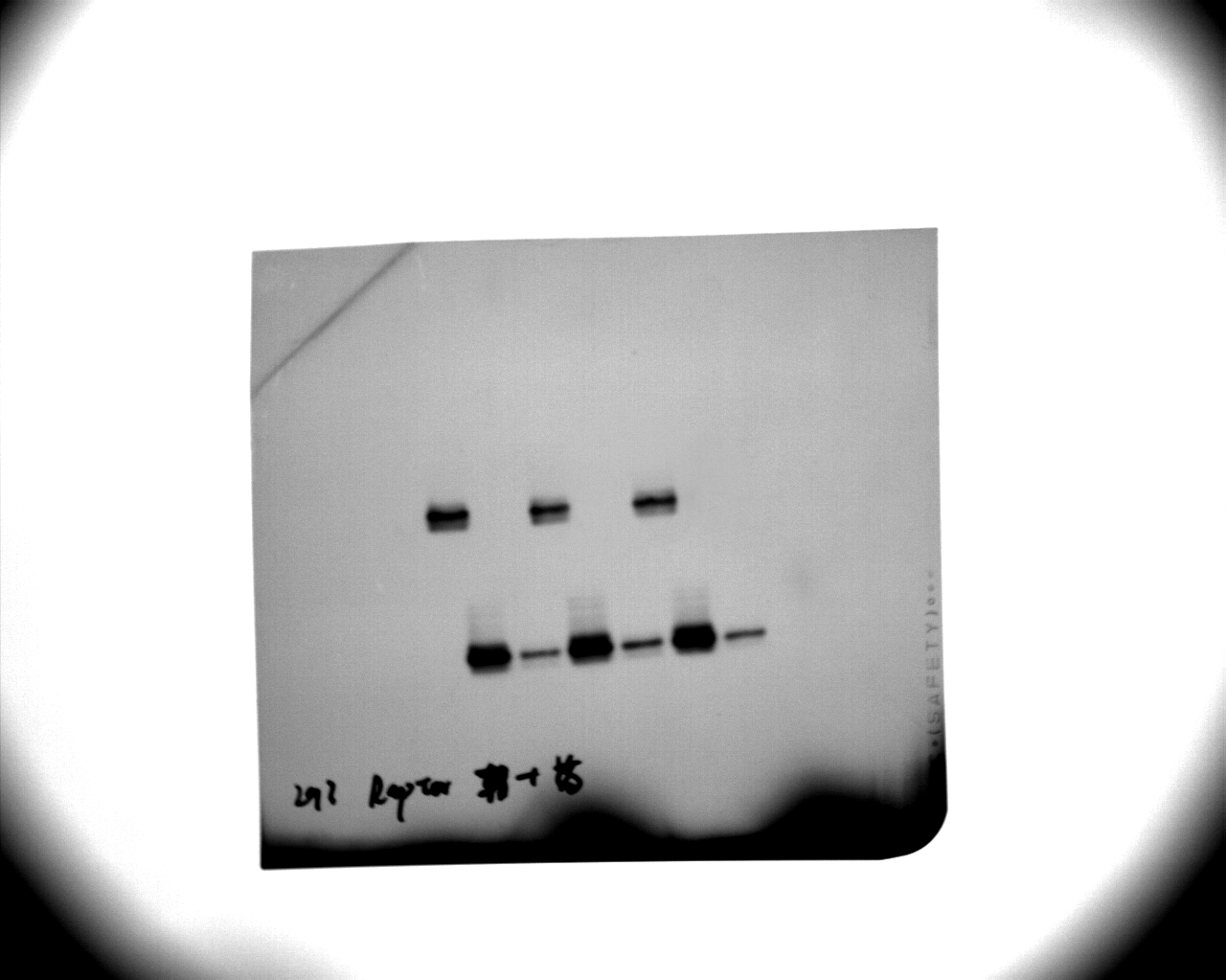
Fig. 5A 293T lysate Raptor



 Fig. 5A 293T lysate RagC



 Fig. 5A 293T lysate actin


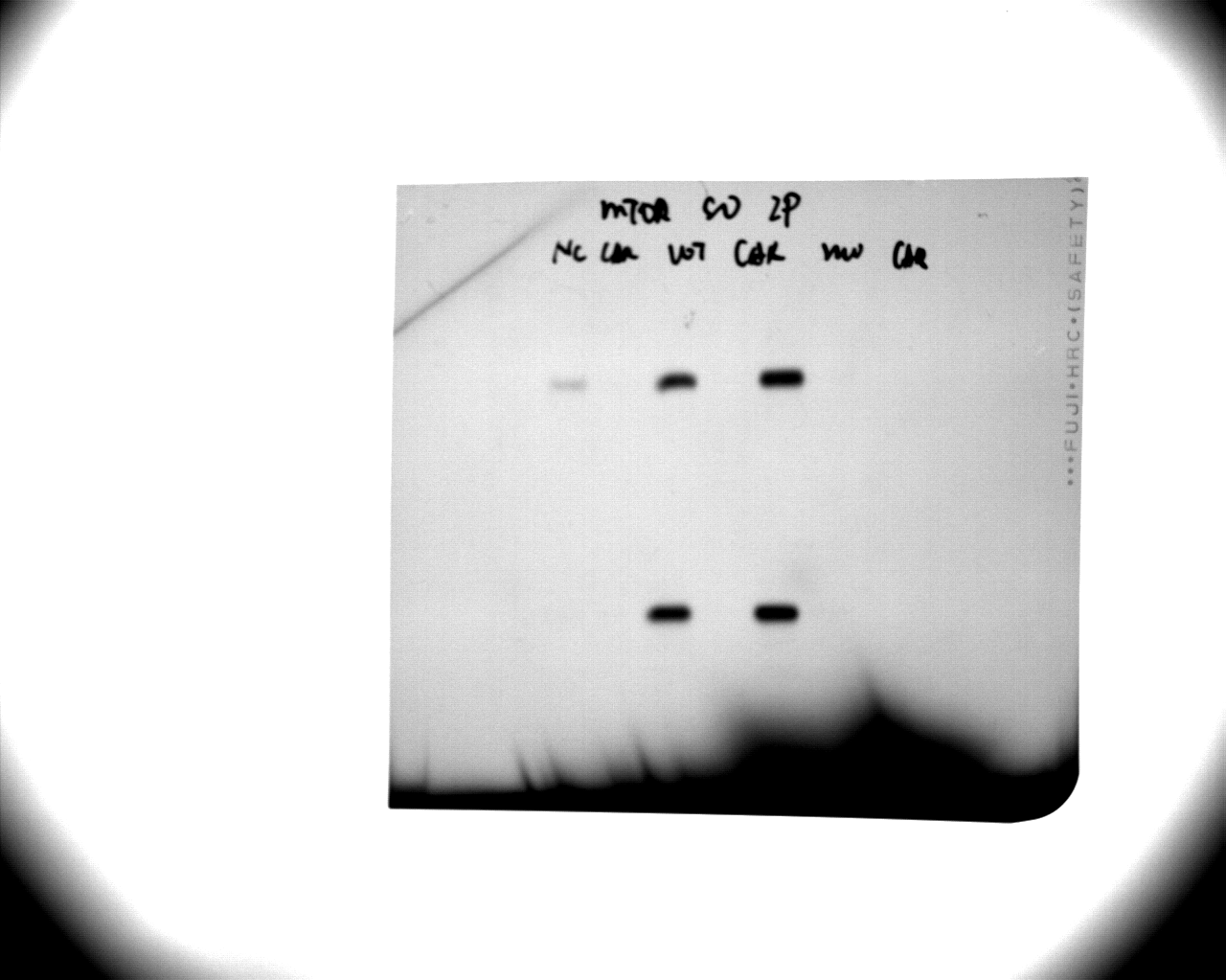
 Fig. 5A SUDHL-4 IP mTOR



 Fig. 5A SUDHL-4 IP Raptor


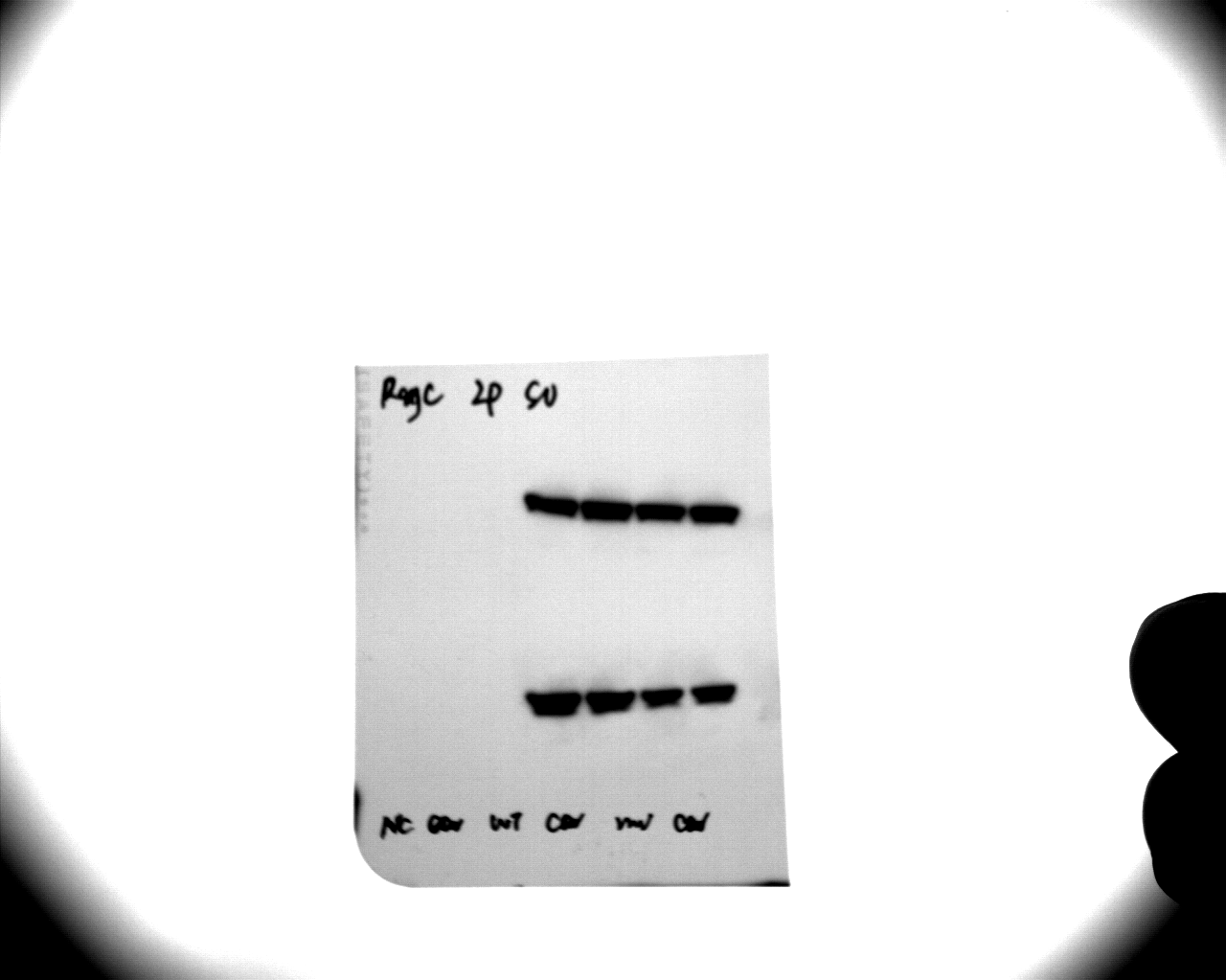
Fig. 5A SUDHL-4 IP RagC


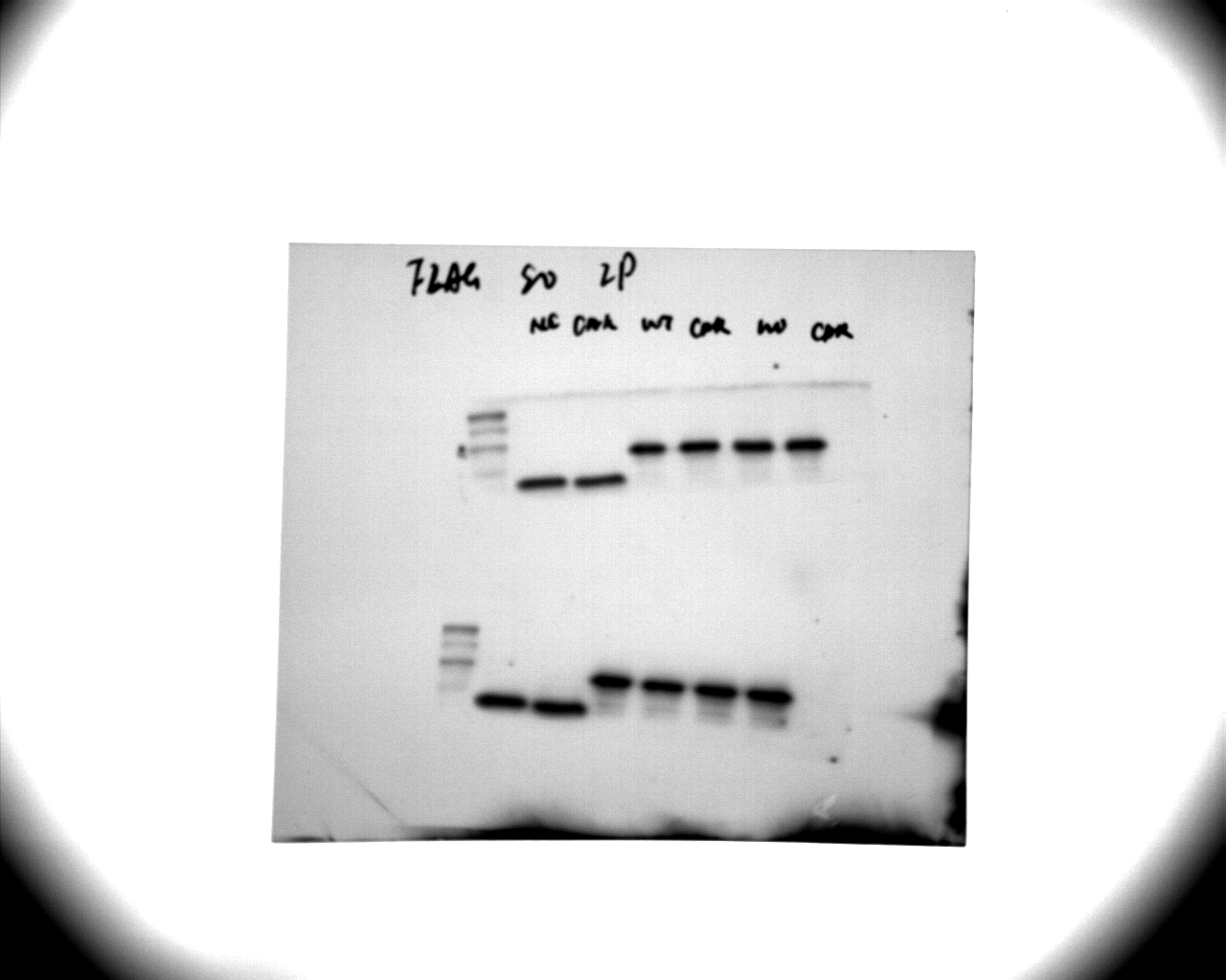
 Fig. 5A SUDHL-4 IP FLAG


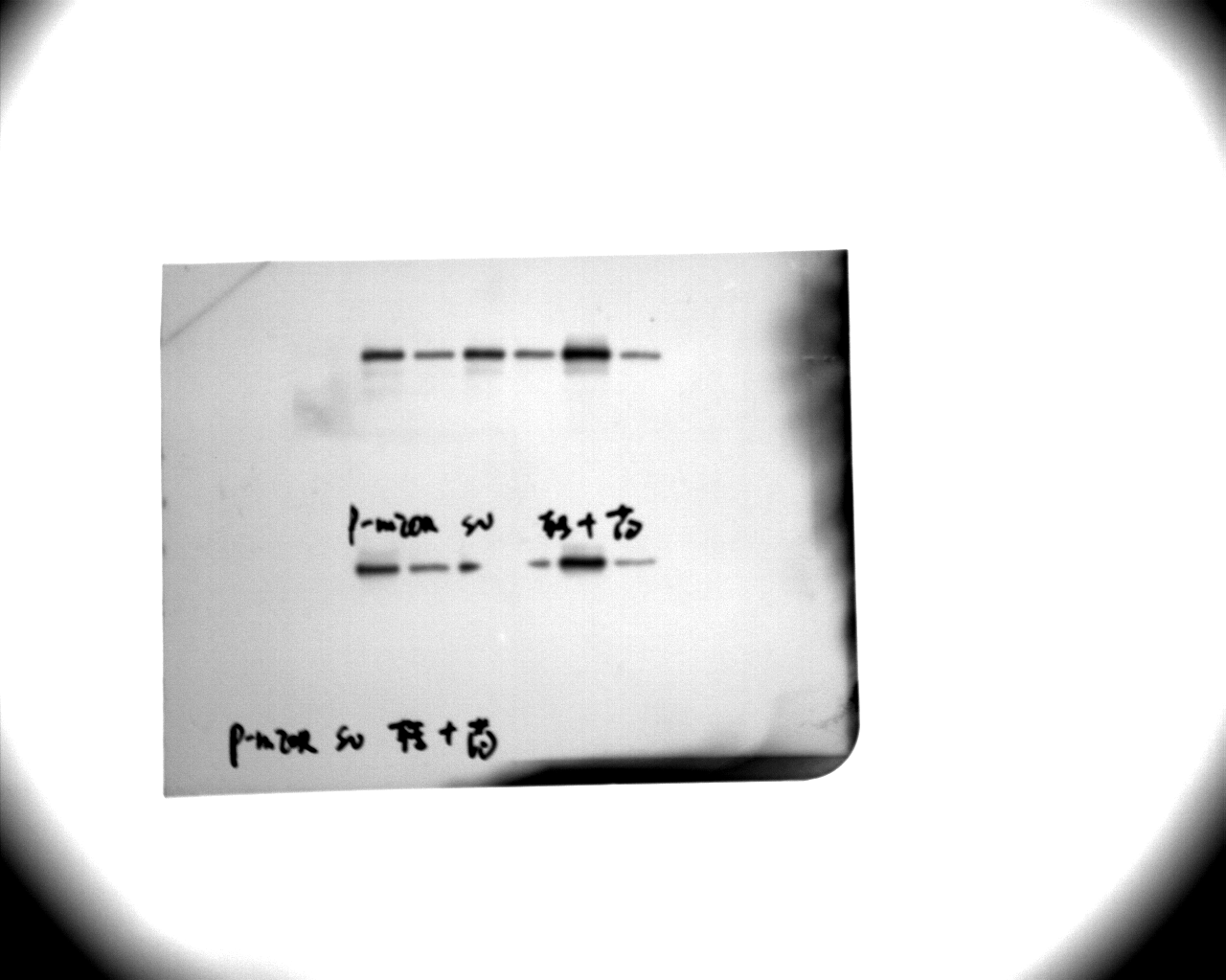
Fig. 5A SUDHL-4 lysate p-mTOR


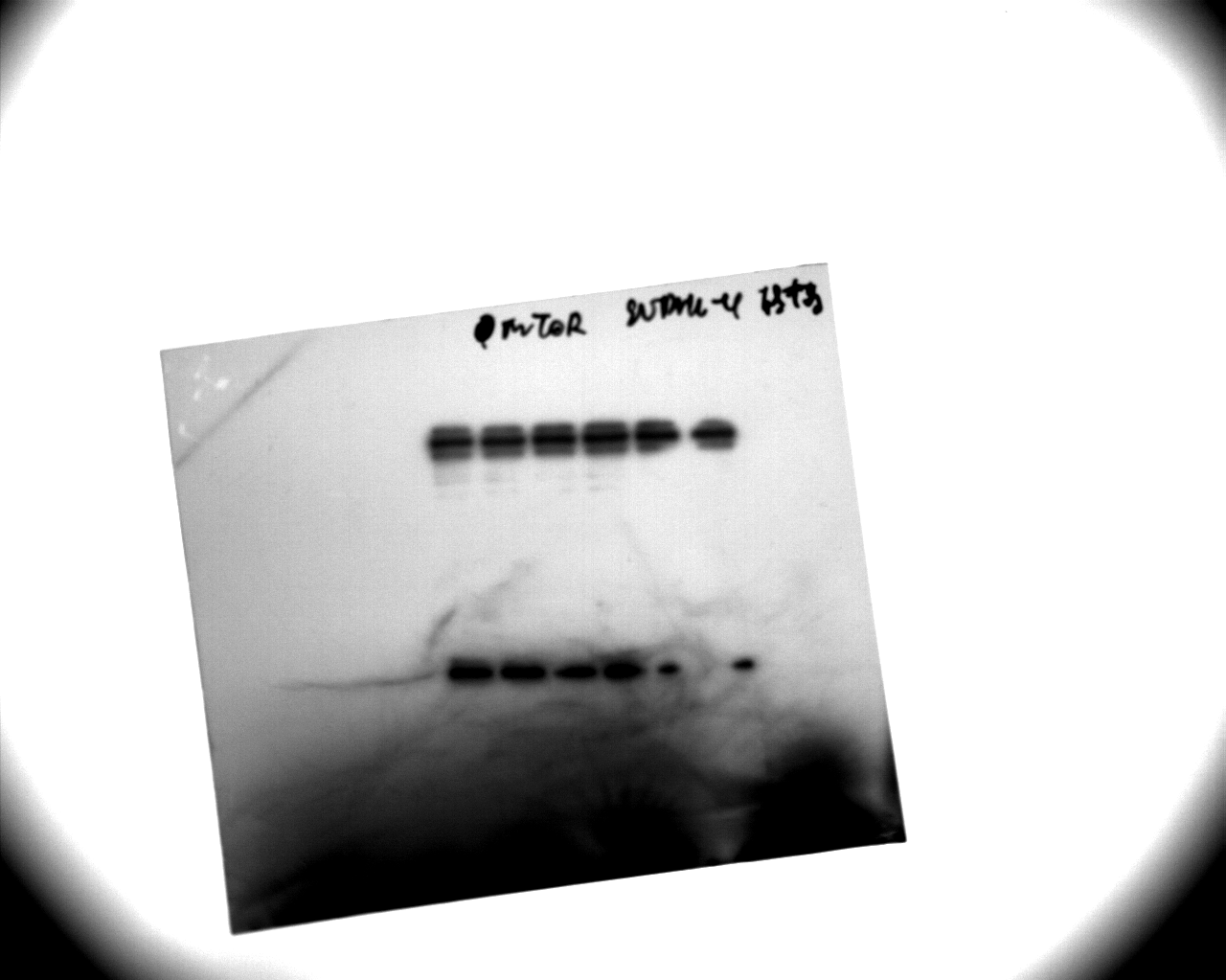
Fig. 5A SUDHL-4 lysate mTOR


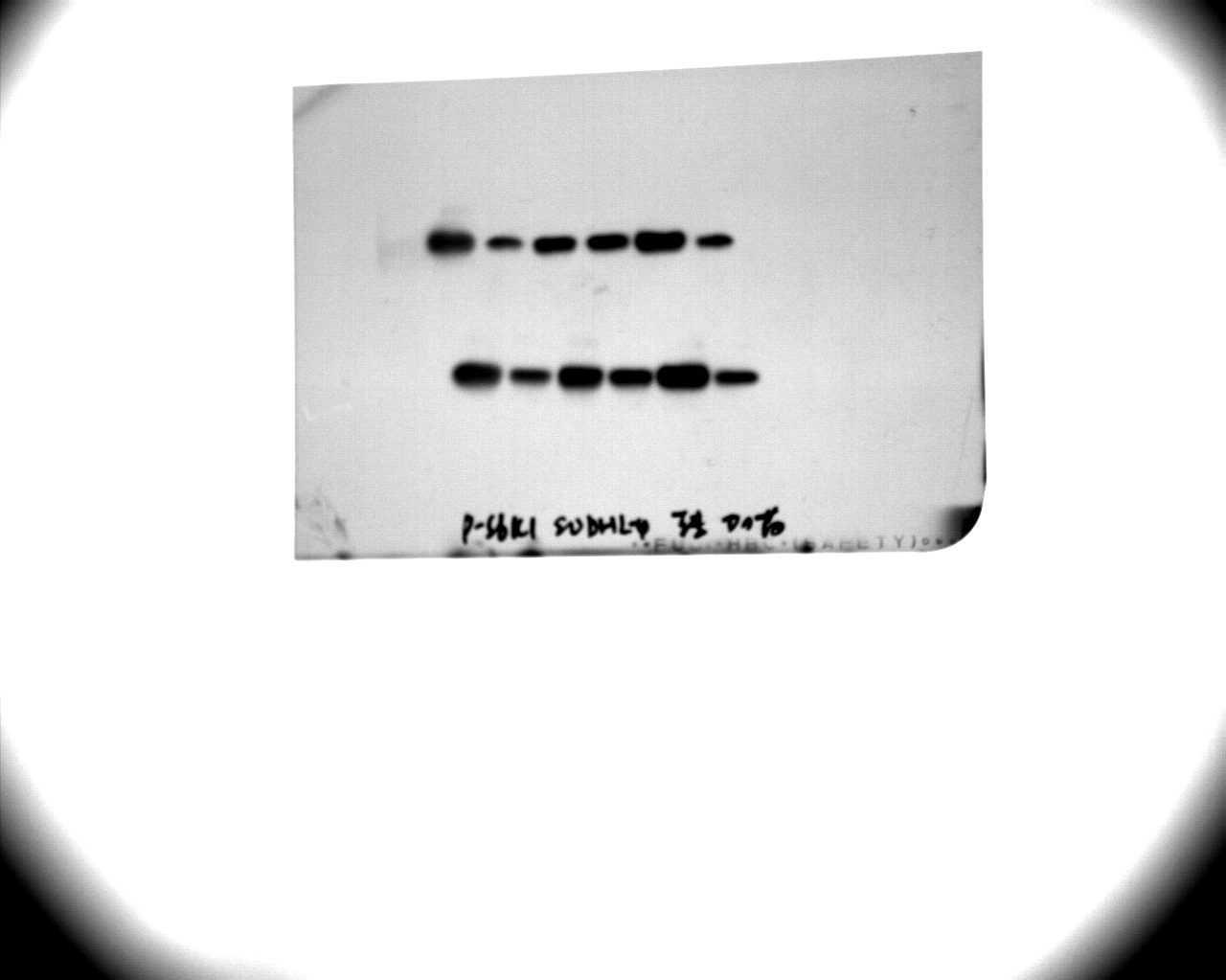
Fig. 5A SUDHL-4 lysate p-S6K1



 Fig. 5A SUDHL-4 lysate S6K1



 Fig. 5A SUDHL-4 lysate Raptor


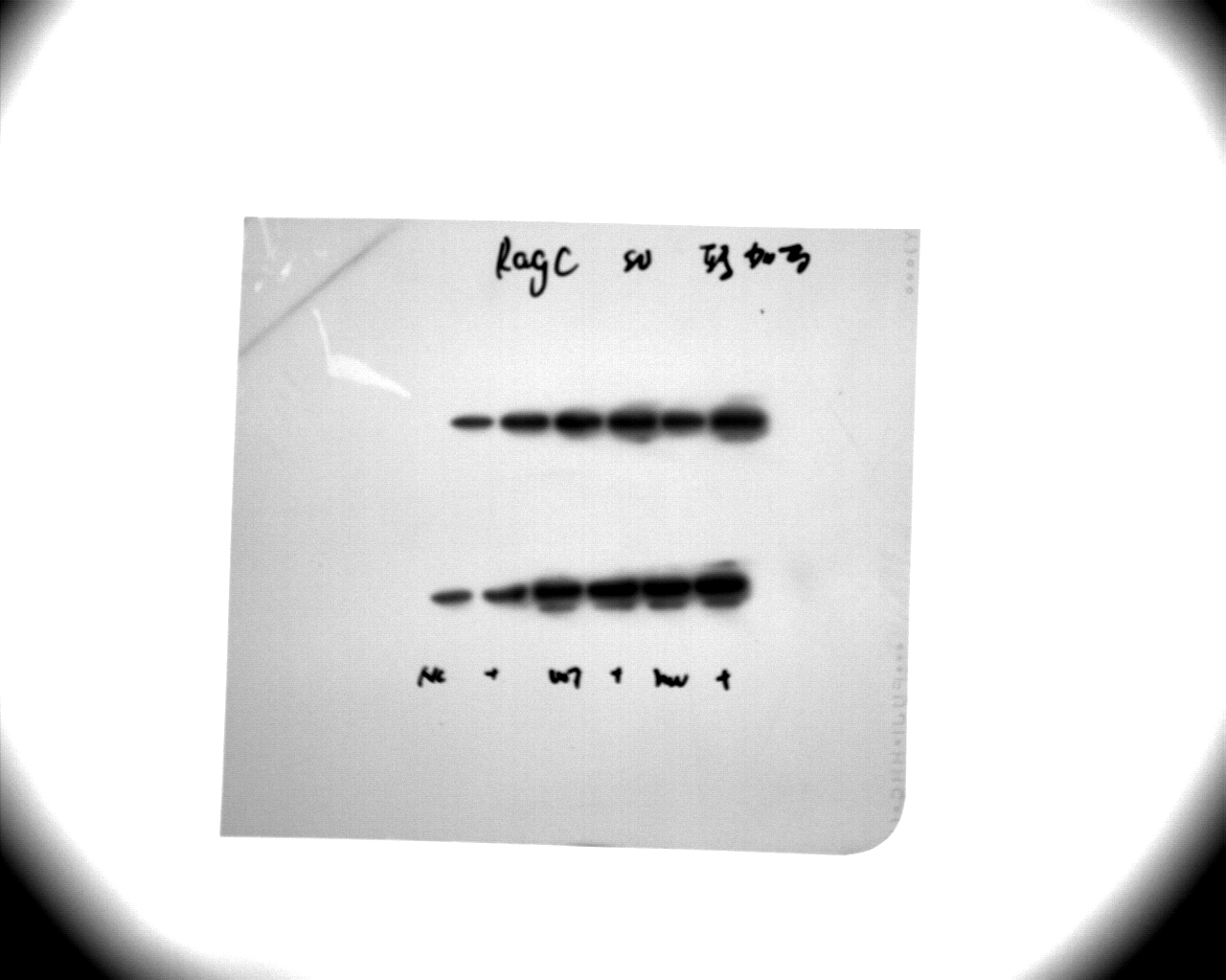
 Fig. 5A SUDHL-4 lysate RagC


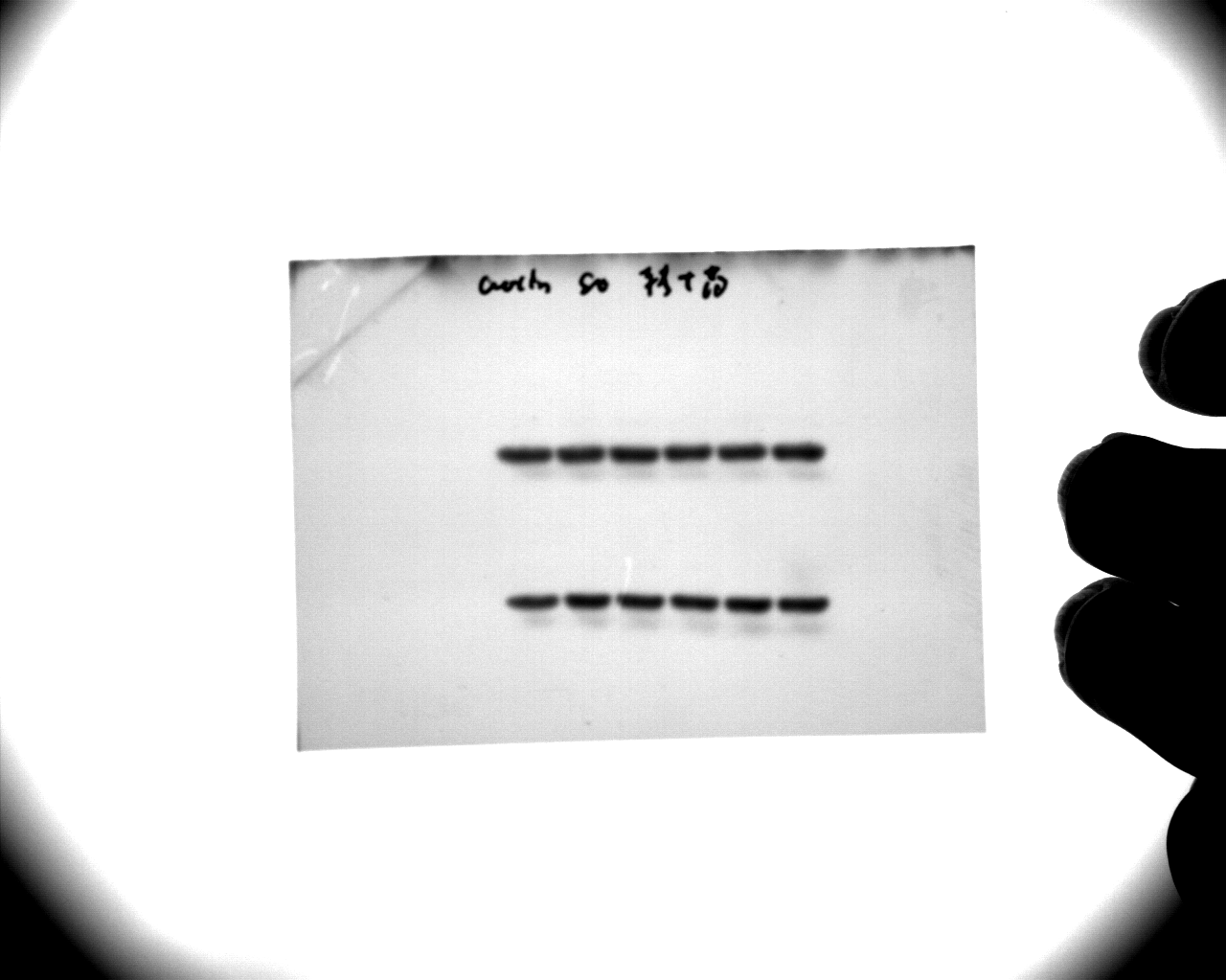
 Fig. 5A SUDHL-4 lysate actin
