# Supplementary material for: Raptor mediates the selective inhibitory effect of cardamonin on RRAGC-mutant B cell lymphoma
Source: BMC Complement Med Ther. 2023 Sep 26;23:336. doi: 10.1186/s12906-023-04166-7 (PMC10521446; doi:10.1186/s12906-023-04166-7)
Supplement: Supplementary file 5 — Supplementary Material 5 [file 12906_2023_4166_MOESM5_ESM.docx]

Supplementary Original western blot images for Figure 5C. Original western blotting for mTOR signalling and Raptor of the cardamonin and everolimus treated RagC ^WT^ and RagC ^T90N^ SUDHL-4 cells. The protein blots are imaged by X-ray film exposure.

Figure 5C


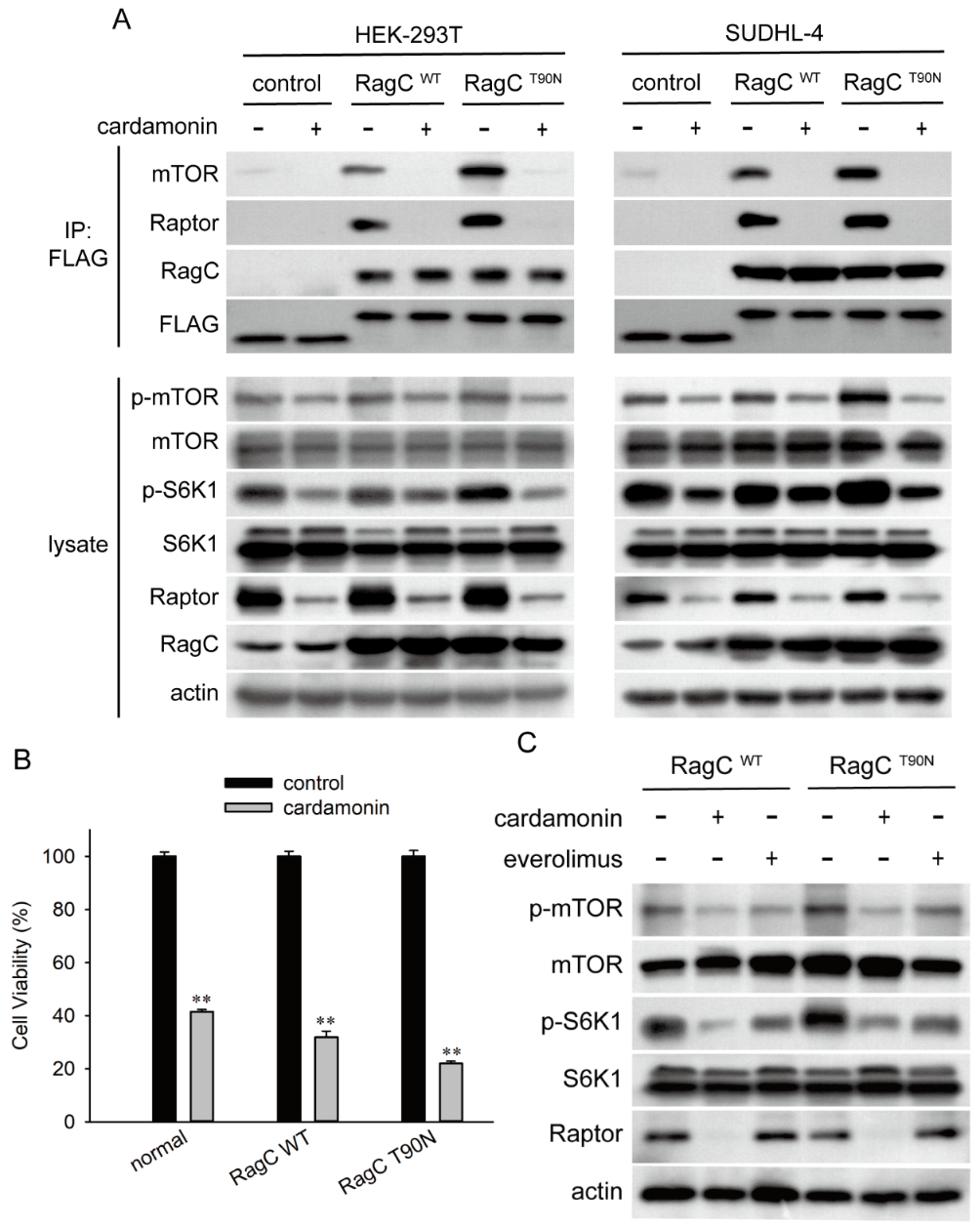



 Fig. 5C p-mTOR



 Fig. 5C mTOR


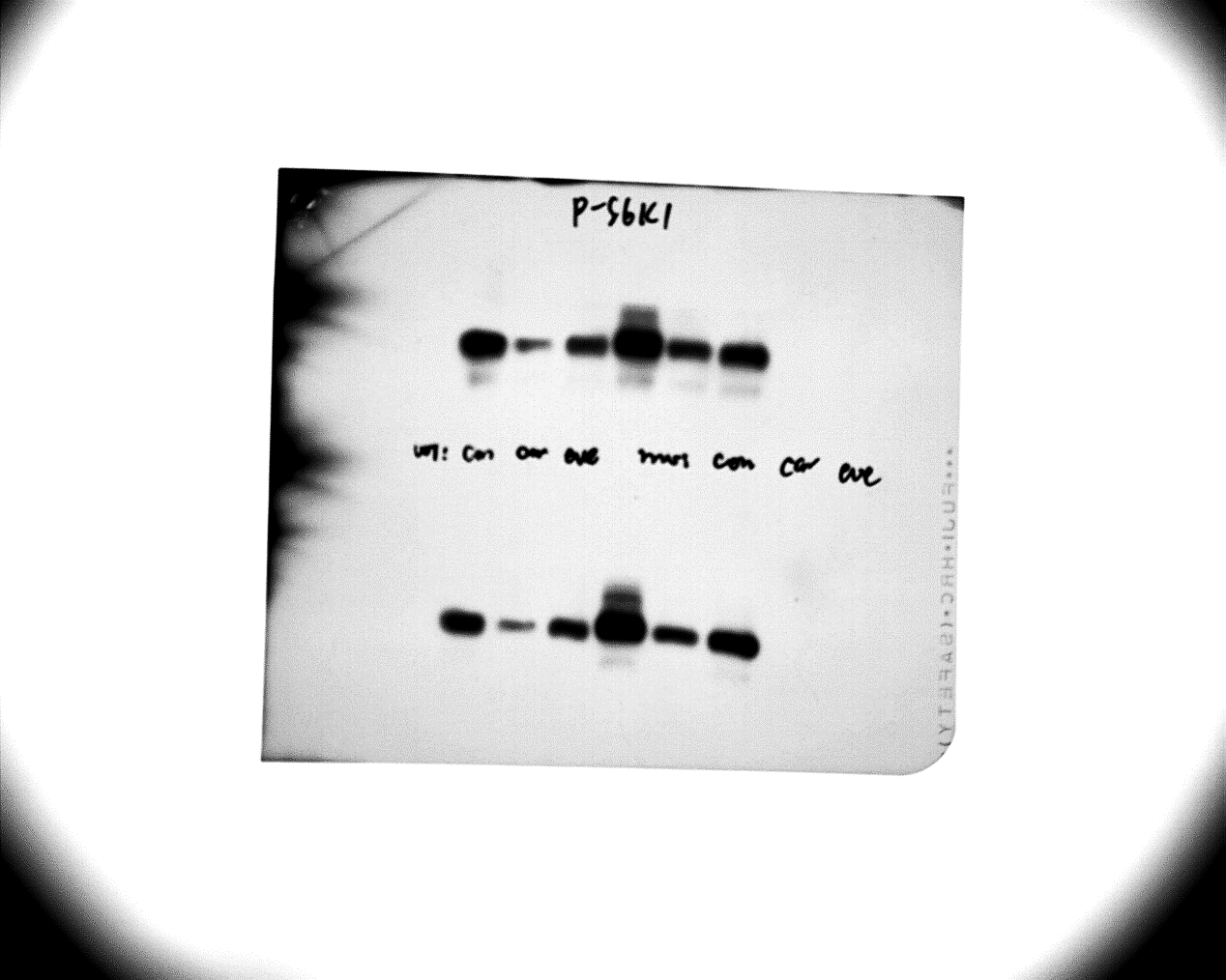
 Fig. 5C p-S6K1


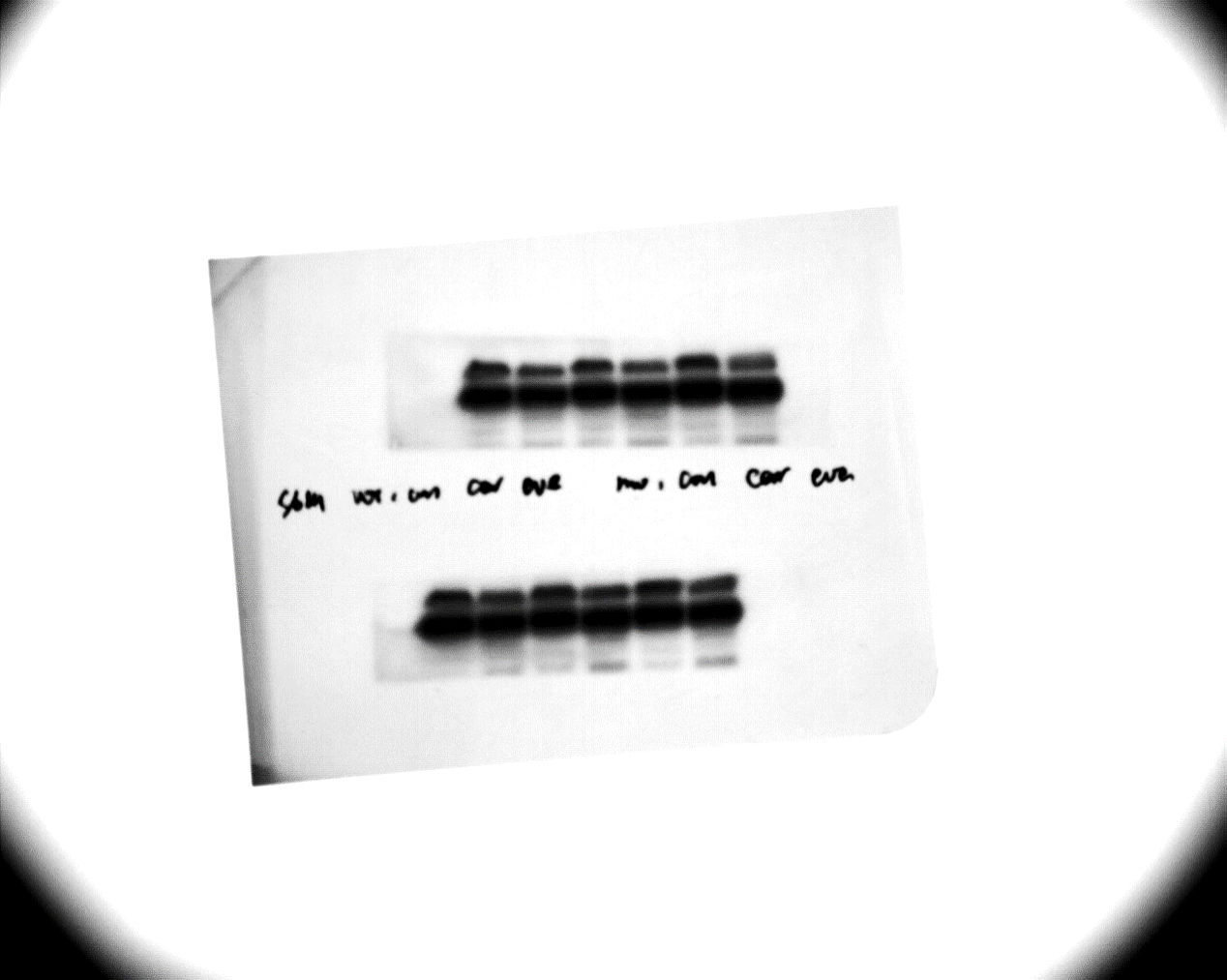
 Fig. 5C S6K1



 Fig. 5C Raptor



 Fig. 5C actin
