# Supplementary material for: Raptor mediates the selective inhibitory effect of cardamonin on RRAGC-mutant B cell lymphoma
Source: BMC Complement Med Ther. 2023 Sep 26;23:336. doi: 10.1186/s12906-023-04166-7 (PMC10521446; doi:10.1186/s12906-023-04166-7)
Supplement: Supplementary file 6 — Supplementary Material 6 [file 12906_2023_4166_MOESM6_ESM.docx]

Supplementary Original western blot images for Figure 6. Original western blotting for mTOR signalling, Raptor and RagC of the FLAG-RagC^WT^, FLAG-RagC^T90N^ transfected SUDHL-4 cells and the Raptor knockdown SUDHL-4 cells in the absent or present of cardamonin. The protein blots are imaged by X-ray film exposure.

Figure 6


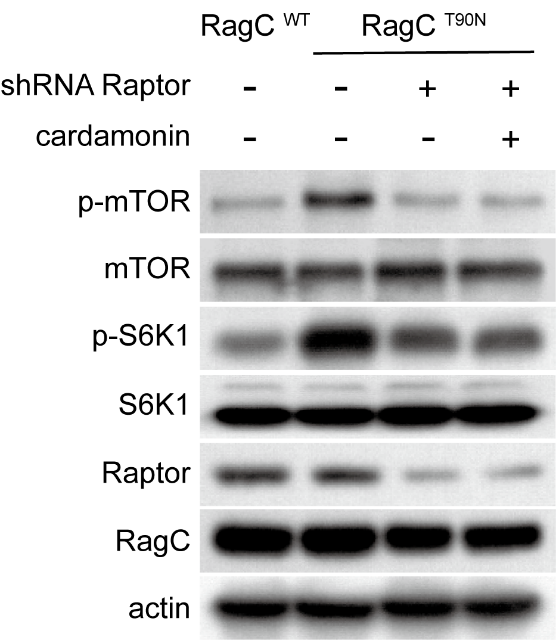


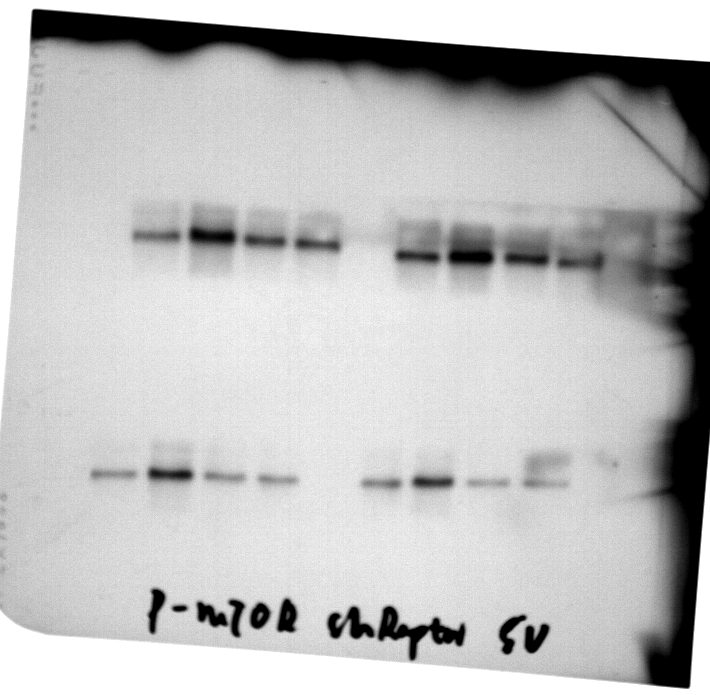
 Fig. 6 p-mTOR


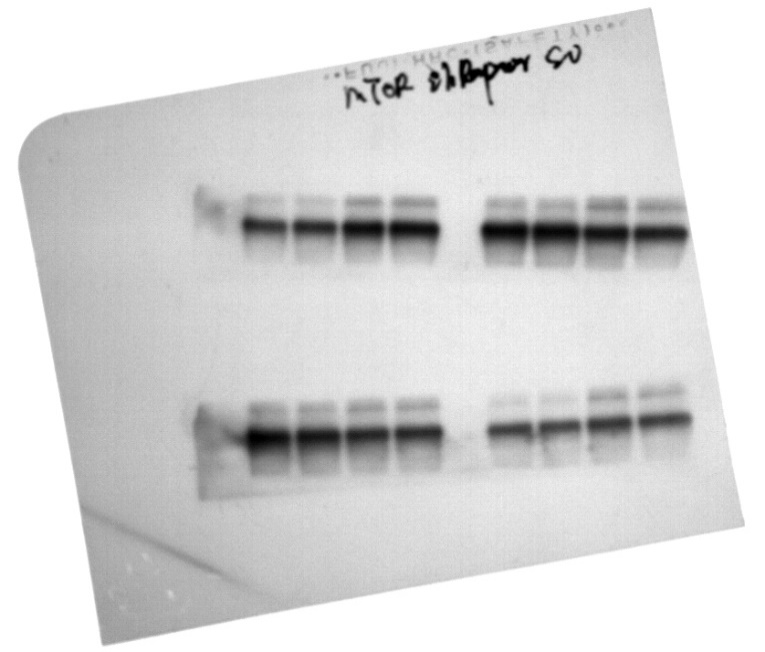
 Fig. 6 mTOR


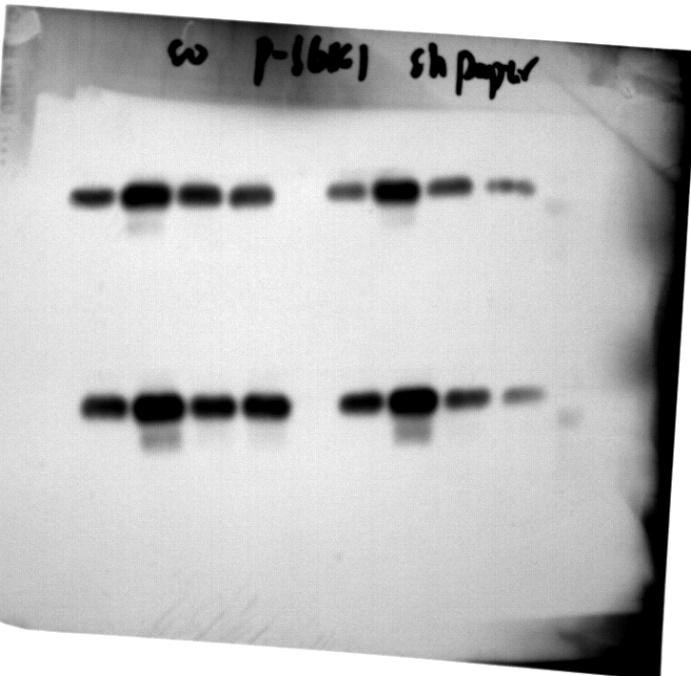
 Fig. 6 p-S6K1


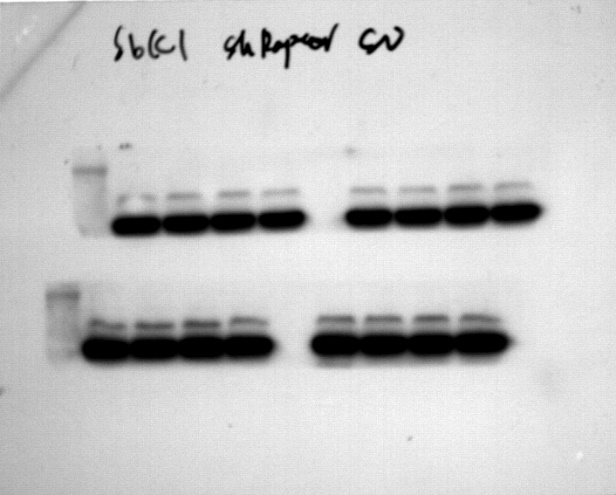
 Fig. 6 S6K1


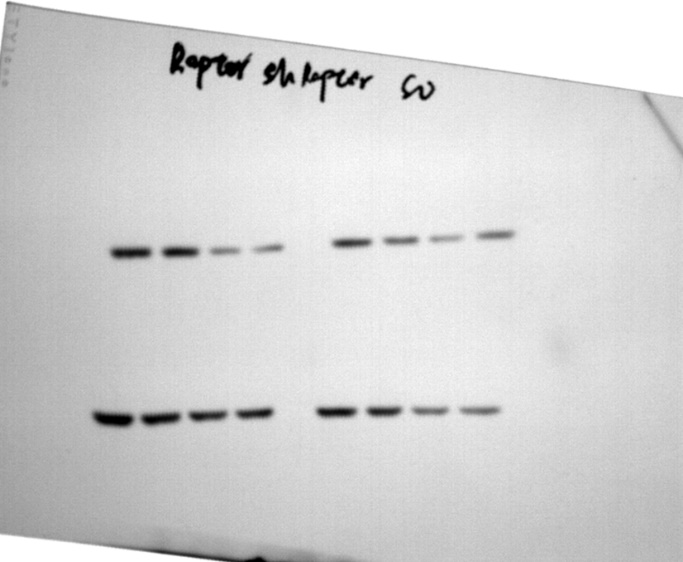
 Fig. 6 Raptor


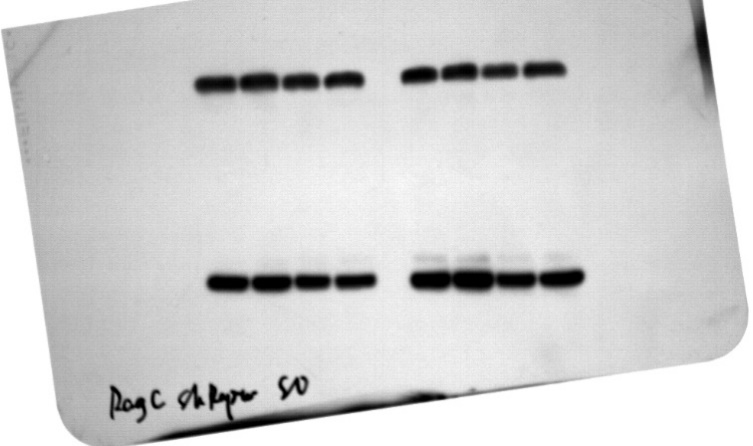
 Fig. 6 RagC


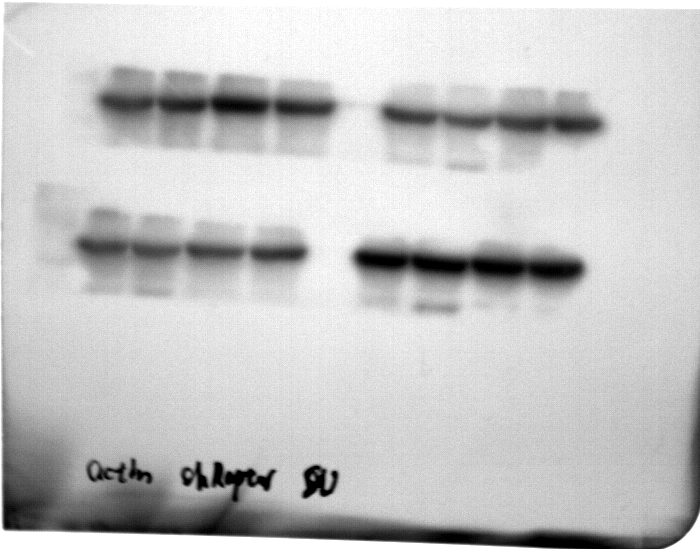
 Fig. 6 actin
